# Ultra-fast-track anesthesia in cardiac surgery: perioperative outcomes, patient selection, and implementation strategies—a narrative review

**DOI:** 10.3389/fcvm.2026.1914025

**Published:** 2026-08-12

**Authors:** Sen Zhao, Jinzhong Yao, Huan Deng, Xi Wang, Meiyi Cui, Mingxing Chen, Jiangtao Meng, Yi Liu, Wei Zhong

**Affiliations:** 1 Department of Anesthesiology, Henan Provincial Chest Hospital, Affiliated Chest Hospital of Zhengzhou University, Zhengzhou, China; 2 Department of Anesthesiology, Shenzhen People’s Hospital, The First Affiliated Hospital, Southern University of Science and Technology, Shenzhen, China; 3 Department of Anesthesiology, The Eighth Affiliated Hospital of Sun Yat-sen University, Shenzhen, China; 4 Department of Human Anatomy, Histology and Embryology, School of Basic Medical Sciences, Zhengzhou University, Zhengzhou, China

**Keywords:** cardiac surgery, cardiovascular surgery, enhanced recovery after surgery, operating-room extubation, patient selection, perioperative outcomes, ultra-fast-track anesthesia

## Abstract

Ultra-fast-track anesthesia (UFTA) is an approach characterized by early endotracheal extubation—typically at the end of surgery or within the first hour after surgery—with the aim of facilitating rapid emergence and early restoration of physiological function in patients undergoing cardiac surgery. Short-acting sedatives, neuromuscular blockade reversal strategies, and regional analgesic techniques have expanded the anesthesiologist's perioperative toolkit and may support selected UFTA pathways; however, they are adjuncts rather than prerequisites, and evidence that any single component independently improves UFTA success remains limited. In carefully selected patients, UFTA has been associated with shorter invasive ventilation and may reduce ICU resource use in selected settings. However, reported effects on ICU or hospital length of stay, pulmonary complications, reintubation, and mortality are heterogeneous, and most available evidence remains susceptible to selection and institutional confounding. This review summarizes UFTA as a multidisciplinary cardiovascular surgery pathway, focusing on perioperative outcomes, patient selection, implementation strategies, and evidence gaps relevant to safe adoption.

## Introduction

Historically, patients recovering from cardiac surgery were kept intubated on prolonged mechanical ventilation and transferred directly to the intensive care unit (ICU) to maintain clinical stability, rather than being extubated immediately after surgery. However, prolonged mechanical ventilation not only increases healthcare costs but also predisposes patients to complications such as ventilator-associated pneumonia and diaphragmatic dysfunction (1, 2). Fast-track cardiac anesthesia, which generally aims to facilitate tracheal extubation within 1–6 h after cardiac surgery, emerged during the 1990s as part of a broader multidisciplinary strategy to improve resource utilization and postoperative recovery (3). As short-acting anesthetic agents, opioid-sparing strategies, neuromuscular blockade management, and perioperative monitoring evolved, they provided additional options for titrated emergence that may support early-extubation pathways (4). At the same time, the enhanced recovery after surgery (ERAS) paradigm gained widespread acceptance, integrating numerous perioperative measures—such as optimized anesthesia, effective analgesia, and early extubation—into routine practice (5). ERAS guidelines for cardiac surgery emphasize minimizing mechanical ventilation duration as a key component of optimal recovery and frame extubation within 6 h after surgery as a fast-track recovery goal for most patients (6). Against this background, the concept of ultra-fast-track anesthesia (UFTA) emerged, referring to extubation performed in the operating room or within one hour after surgery (7).

In recent years, multiple studies have evaluated UFTA in adult and pediatric cardiac surgery. The most frequently reported finding is shorter invasive ventilation; reported effects on ICU stay, hospital stay, costs, mortality, and other adverse outcomes are heterogeneous and may be influenced by patient selection and study design (8–10). Some studies report fewer postoperative pulmonary complications, although this finding is not uniform (11). In pediatric patients with congenital heart disease, selected cohorts have reported earlier extubation and shorter ICU or hospital stays, but procedure complexity and selection criteria limit generalizability (10).

Accordingly, this review addresses not simply whether extubation can be performed earlier, but which patients may benefit, under what procedural and institutional conditions UFTA can be considered, and whether its benefits extend beyond shorter invasive ventilation. Compared with prior fast-track or ERAS reviews, this review emphasizes the strict within-1-h UFTA definition, outcome hierarchy, dynamic patient selection, and rescue-capacity requirements that determine whether early extubation is clinically meaningful.

## Methods for literature identification and narrative synthesis

This article is a narrative review. To improve the transparency and breadth of literature identification, we undertook a structured, concept-based search update during revision. PubMed (including MEDLINE-indexed records) was searched on July 21, 2026, and Embase (Embase.com), Web of Science Core Collection (Clarivate), CINAHL (EBSCOhost), and the Cochrane Library (Cochrane Database of Systematic Reviews and CENTRAL) were searched on July 22, 2026, from database inception to the respective search date. English-language reports were eligible. Exact database-specific strategies, platforms, dates, and retrieval counts are provided in Supplementary File 1.

The search combined cardiac-surgery terms with seven concept domains: (1) UFTA and operating-room, on-table, intraoperative, immediate, or early extubation; (2) anesthetic techniques and short-acting, opioid-sparing, or multimodal regimens; (3) quantitative neuromuscular monitoring and reversal; (4) enhanced-recovery and fast-track pathways or protocols; (5) regional analgesia; (6) post-extubation respiratory support; and (7) patient selection, extubation readiness, delayed extubation, pathway failure, and implementation. Named agents, including remimazolam, sugammadex, and neostigmine, were embedded within broader concept blocks and did not determine eligibility.

Eligible sources included randomized and nonrandomized clinical studies, systematic reviews, meta-analyses, and professional guidance concerning adult or pediatric cardiac surgery. Sources were eligible if they addressed strict UFTA (operating-room or within-1-hour extubation), 1–6-hour fast-track extubation as contextual evidence, relevant perioperative pathways or techniques, or patient selection, readiness, failure, rescue, and implementation. Smaller observational studies and case series were considered for emerging techniques, uncommon procedures, or special populations. We excluded noncardiac surgery, peripheral vascular or abdominal aortic surgery, transcatheter-only and nonsurgical critical-care populations without separable cardiac-surgical data, nonclinical studies, duplicate or inferior reports, nondata records, and studies without relevant extubation, ventilation, respiratory-safety, ICU-use, recovery, selection, or implementation information. Sources outside these clinical eligibility criteria could be retained only for clearly identified background, mechanistic, or professional-guidance context and were not used as direct evidence for cardiac-surgical outcomes.

Across the seven overlapping search domains, 11,408 records were retrieved. Within-database unioning contributed 7,903 records to cross-database deduplication; identifier-based deduplication yielded 5,075 candidate records, and adjudication of 730 exact normalized-title duplicates yielded 4,345 report-level records for title-and-abstract screening. Screening excluded 1,426 records and retained 2,919 as a broad potentially relevant evidence pool. For focused source verification, the authors confirmed full-text review of 117 prioritized PubMed records; this verification subset and the final 73-source citation set overlap but are not nested counts. Because this was a narrative review, these 2,919 records were not treated as an exhaustively assessed systematic-review inclusion set. Sources for narrative synthesis were purposively selected according to relevance to the review questions, evidence directness, study design, completeness of outcome reporting, coverage of predefined domains, and avoidance of redundant reports. After final EndNote reconciliation, the manuscript cites 73 unique sources. Forty-six were linked by exact title to the reconstructed five-database set: 45 met the clinical eligibility criteria, whereas one MitraClip report was retained solely as indirect pharmacological context and not as direct cardiac-surgical evidence. The remaining 27 non-linked sources were manually verified by the authors. A PRISMA-style narrative evidence-selection flow diagram, title-and-abstract exclusion reasons, and full database-specific search strategies are provided in Supplementary File 1 (Supplementary Figure S1, Supplementary Tables S3, S4, and Supplementary Appendix A, respectively).

Evidence was prioritized by design and directness: systematic reviews and randomized trials for comparative outcomes; multicenter, registry, and large comparative cohorts for safety, uncommon events, generalizability, and resource use; professional guidance for monitoring and implementation; and smaller studies for emerging techniques or special populations. Evidence was classified as direct strict-UFTA evidence, indirect 1–6-hour fast-track evidence, or indirect pathway-component evidence. Pathway-component studies were not interpreted as showing that an individual drug, block, or respiratory-support technique independently improved UFTA success. No protocol was registered, screening was not performed as duplicate independent screening, and no formal risk-of-bias assessment, certainty grading, or meta-analysis was undertaken; the structured record improves transparency without reclassifying this narrative review as a systematic review.

## Definition and concept of ultra-fast-track anesthesia

UFTA generally refers to removing the endotracheal tube immediately at the end of surgery or within 1 h after surgery, with extubation often performed directly in the operating room (8). UFTA relies primarily on precise anesthetic titration, coordinated multidisciplinary care, and readiness assessment; short-acting agents and other adjuncts may be incorporated when clinically appropriate (12). For example, volatile anesthetics may be reduced or temporarily discontinued during cardiopulmonary bypass, and neuromuscular blocking agents should be titrated according to surgical requirements, rewarming progress, and quantitative neuromuscular monitoring; short-acting opioids are used for analgesia to minimize residual anesthetic effects (7). Anesthetic agents are typically tapered off before skin closure so that the patient begins emerging from anesthesia and meets extubation criteria by the end of surgery. In addition, regional techniques may be incorporated as one component of multimodal analgesia in selected patients, but they are not required for UFTA and should not be assumed to independently improve extubation outcomes (13, 14). Across prior reports, these elements are implemented as part of a protocolized perioperative pathway rather than as isolated anesthetic maneuvers (12, 15–17).

For clarity, this review distinguishes four related but non-identical concepts: operating-room extubation, UFTA defined as extubation in the operating room or within 1 h after surgery, fast-track extubation within 1–6 h, and delayed extubation beyond 6 h (3, 8). Studies comparing intraoperative extubation with extubation within 6 h should therefore be interpreted as UFTA vs. conventional fast-track care rather than UFTA vs. prolonged postoperative ventilation.

## Comparison between UFTA and conventional anesthetic strategies

The key difference between UFTA and conventional anesthesia is the perioperative goal. Conventional practice often uses higher anesthetic/analgesic doses and maintains postoperative intubation with ICU ventilation, whereas UFTA optimizes drug selection and timing to minimize residual effects and enable extubation immediately after surgery, often in the operating room (17). The principal reported process effect is shorter invasive ventilation, whereas effects on ICU stay, hospital stay, complications, mortality, and cost vary across studies (8). Reported outcomes are summarized cautiously in Table 1.

**Table 1:** Outcome-domain summary of reported findings associated with UFTA and conventional postoperative ventilation strategies.

Outcome domain and endpoint	UFTA	Comparator strategy	Note
Process outcome: postoperative extubation time	Extubation in the operating room or within 1 h after surgery	Heterogeneous strategies including ICU extubation, conventional fast-track extubation (≤6 h), or delayed postoperative ventilation	UFTA has been associated with shorter invasive ventilation in selected studies, but definitions and comparator strategies vary. (5, 8, 18)
Process outcome: ICU length of stay	Often shorter in selected cohorts	Variable	The magnitude of ICU-stay reduction is heterogeneous and may reflect pathway and selection effects. (38, 39)
Process outcome: total hospital length of stay	May be shorter	Variable	Reported reductions vary; adult and pediatric estimates should not be treated as interchangeable. (10, 39)
Patient-centered safety outcome: reintubation	Generally low in selected patients	Generally low	No consistent difference has been established; strict selection strongly influences observed rates. (15, 39–41, 67, 68)
Patient-centered safety outcome: pulmonary complications	Possible reduction in some cohorts	Variable	Evidence is predominantly observational and is not uniform across procedures or populations. (8, 15, 40, 41, 69)
Recovery or neurocognitive outcome: postoperative delirium	Possible association with lower delirium in selected cohorts	Variable	An observational association does not establish neuroprotection or causality. (70)
Patient-centered safety outcome: major adverse events	Possible reduction in selected cohorts	Variable	Observed differences may partly reflect patient selection and institutional practice. (8)
Patient-centered safety outcome: short-term mortality (30-day, operative, or in-hospital, as defined by each study)	No consistent signal of increased mortality	Uncertain; no consistent signal of difference	No consistent signal of increased mortality has been reported in selected cohorts; however, available studies may be underpowered to detect small mortality differences. (15, 38, 40, 42)
Resource-use outcome: hospitalization costs	May be lower when ICU use decreases	Variable	Economic effects are center- and pathway-dependent; no universal percentage reduction is established. (39, 69)

Comparator strategies were heterogeneous across studies and included ICU extubation, conventional fast-track extubation (≤6 h), or delayed postoperative ventilation. This outcome categorization is conceptual and was not derived from a formal meta-analytic framework (5, 15, 18).

For interpretation, reported outcomes should be separated into process outcomes (18) (extubation time, ventilation duration, ICU or hospital stay, and cost), patient-centered safety outcomes (reintubation, respiratory failure, pneumonia, stroke, acute kidney injury, low cardiac output, bleeding, mortality, and readmission), and recovery outcomes (mobilization, oral intake, quality of recovery, functional recovery, and patient satisfaction) (5, 15). Lack of a statistically significant difference in safety outcomes should not be interpreted as proof of equivalence.

## Pharmacological and technical adjuncts within UFTA pathways

UFTA depends on coordinated anesthetic titration, appropriate neuromuscular management, analgesia, extubation-readiness assessment, and postoperative rescue capacity (12). Individual drugs, reversal agents, and regional techniques should be interpreted as optional adjuncts within a perioperative pathway rather than prerequisites or independent determinants of successful operating-room extubation. Differences between conventional and UFTA-compatible approaches are summarized in Table 2; these components should be interpreted in the context of pathway and neuromuscular-monitoring evidence (19).

**Table 2:** Potential pharmacological and technical components of UFTA pathways.

Category	Potential limitation in non-optimized pathways	Optional pathway-compatible approach	Rationale
Sedatives	Long-acting or insufficiently titrated sedative regimens	Carefully titrated sedatives selected to minimize residual effects; short-acting options may be used when appropriate	May facilitate titration and hemodynamic management, but no sedative has independently established UFTA success. (22)
Analgesics	Opioid-dominant regimens with greater potential for residual respiratory depression	Carefully titrated short-acting opioids, with optional regional analgesia as part of multimodal care	May reduce residual opioid effects and improve analgesia; block-specific evidence for UFTA success remains limited (71)
Neuromuscular blockade reversal	Reversal without quantitative monitoring or before adequate recovery	Quantitative monitoring with neostigmine or sugammadex selected according to the blocker, depth, and clinical context	Can support timely recovery; neither the reversal agent nor monitoring alone determines UFTA success. (9)
Airway management	Postoperative extubation in ICU	Operating-room extubation with clinically indicated oxygen therapy or HFNC	May support gas exchange after extubation; cardiac-specific comparative outcome evidence remains limited. (72, 73)
Regional Analgesia Techniques	Systemic opioid-dominant analgesia or limited/no regional analgesia	Selected regional blocks within multimodal analgesia	May reduce pain and opioid requirements; bleeding risk, anticoagulation, local-anesthetic dose, incision type, local expertise, and patient selection remain important. (14, 27, 71)

These optional components are illustrative rather than prescriptive. No single sedative, opioid, reversal agent, regional technique, or respiratory-support strategy is required for successful UFTA or has independently established UFTA success.

## New sedatives and neuromuscular blockade reversal agents

### Remimazolam

Remimazolam is a novel ultra-short-acting intravenous benzodiazepine sedative that is metabolized by tissue non-specific esterases, with pharmacokinetic properties that may be less dependent on conventional hepatic metabolism (20). It has a specific antagonist, flumazenil, which overcomes limitations of traditional benzodiazepines such as delayed emergence and residual sedation (21). In terms of hemodynamic effects, remimazolam is associated with less pronounced vasodilation than propofol, making it potentially useful in cardiac surgery patients vulnerable to induction-related hypotension, although direct evidence for improved UFTA success remains limited (22). Small case reports and case series suggest that remimazolam may provide acceptable hemodynamic stability in patients with advanced heart failure or low left ventricular function. However, evidence that it directly facilitates early extubation remains limited (23). At present, remimazolam should be regarded as a hemodynamically attractive sedative option rather than an evidence-established facilitator of operating-room extubation; further studies are needed to define optimal dosing and integration with multimodal UFTA pathways.

### Sugammadex

Neuromuscular blockade may facilitate surgical exposure during cardiac surgery; however, residual neuromuscular blockade can impair postoperative respiratory function and delay safe extubation (19). Sugammadex, a selective neuromuscular blockade reversal agent, acts by encapsulating steroidal neuromuscular blocking agents such as rocuronium and vecuronium, thereby enabling rapid reversal when appropriately dosed and guided by quantitative neuromuscular monitoring (24). Compared with neostigmine, sugammadex can provide more rapid and predictable reversal of rocuronium- or vecuronium-induced blockade and avoids cholinergic adverse effects, although incomplete reversal and other adverse events remain possible; quantitative monitoring is still required. A randomized controlled trial of 60 children undergoing cardiac surgery found that sugammadex shortened time to extubation by approximately 94 min (31.0 vs. 125.2 min) and reduced postoperative atelectasis (0% vs. 20%) (9). No serious adverse events were reported in the available adult trial, although the study was not powered to establish equivalence for rare safety outcomes (25). Some studies have reported lower arterial partial pressure of carbon dioxide (PaCO_2_) after extubation, suggesting more complete recovery of respiratory muscle function (26). Its role in UFTA pathways is best conceptualized as reducing residual neuromuscular blockade and respiratory compromise, rather than as a standalone determinant of successful UFTA. Quantitative train-of-four monitoring remains important, and sugammadex use cannot replace adequate rewarming, acid-base correction, and direct assessment of respiratory and hemodynamic readiness.

### Regional analgesia techniques

Exclusive reliance on high-dose opioids for analgesia can depress respiratory drive and impede early extubation. Regional analgesia may therefore be incorporated into selected opioid-sparing pathways, but its contribution should be interpreted according to incision type, anticoagulation status, and institutional expertise. Meta-analytic evidence suggests that ESPB may reduce postoperative pain and opioid consumption after sternotomy, whereas evidence from OPCAB on-table extubation cohorts describes feasibility in selected patients; together, these sources do not prove that any single block technique independently increases UFTA success (14, 17). For minimally invasive thoracotomy or thoracoscopic approaches, serratus anterior plane block, intercostal nerve block, or related fascial-plane techniques may be more anatomically relevant, but procedure-specific evidence remains limited. In cardiac surgery, block choice must account for systemic heparinization, coagulation status, antiplatelet exposure, puncture-site bleeding risk (27), total local-anesthetic dose, surgical approach, and institutional experience; analgesic benefits should not be assumed to translate directly into reductions in major postoperative complications.

### Post-extubation respiratory support strategies

In UFTA protocols, extubation does not mark the end of respiratory management. High-flow nasal cannula (HFNC) oxygen therapy delivers warmed and humidified oxygen at high flow rates, improves oxygenation, reduces work of breathing, washes out upper-airway dead space, and provides a mild positive end-expiratory pressure effect (28, 29). However, available evidence is indirect for strict adult cardiac UFTA pathways, and HFNC or NIV should be viewed as post-extubation support or rescue options rather than routine components of the UFTA definition. A randomized JAMA trial compared HFNC with noninvasive ventilation in hypoxemic patients after cardiothoracic surgery, not specifically in patients managed with operating-room extubation (28). HFNC should therefore be described as selected post-extubation support, treatment for post-extubation hypoxemia, or rescue therapy before reintubation rather than as routine care for all UFTA patients.

## Patient selection across the perioperative pathway and dynamic go/no-go reassessment

**Figure 1:**
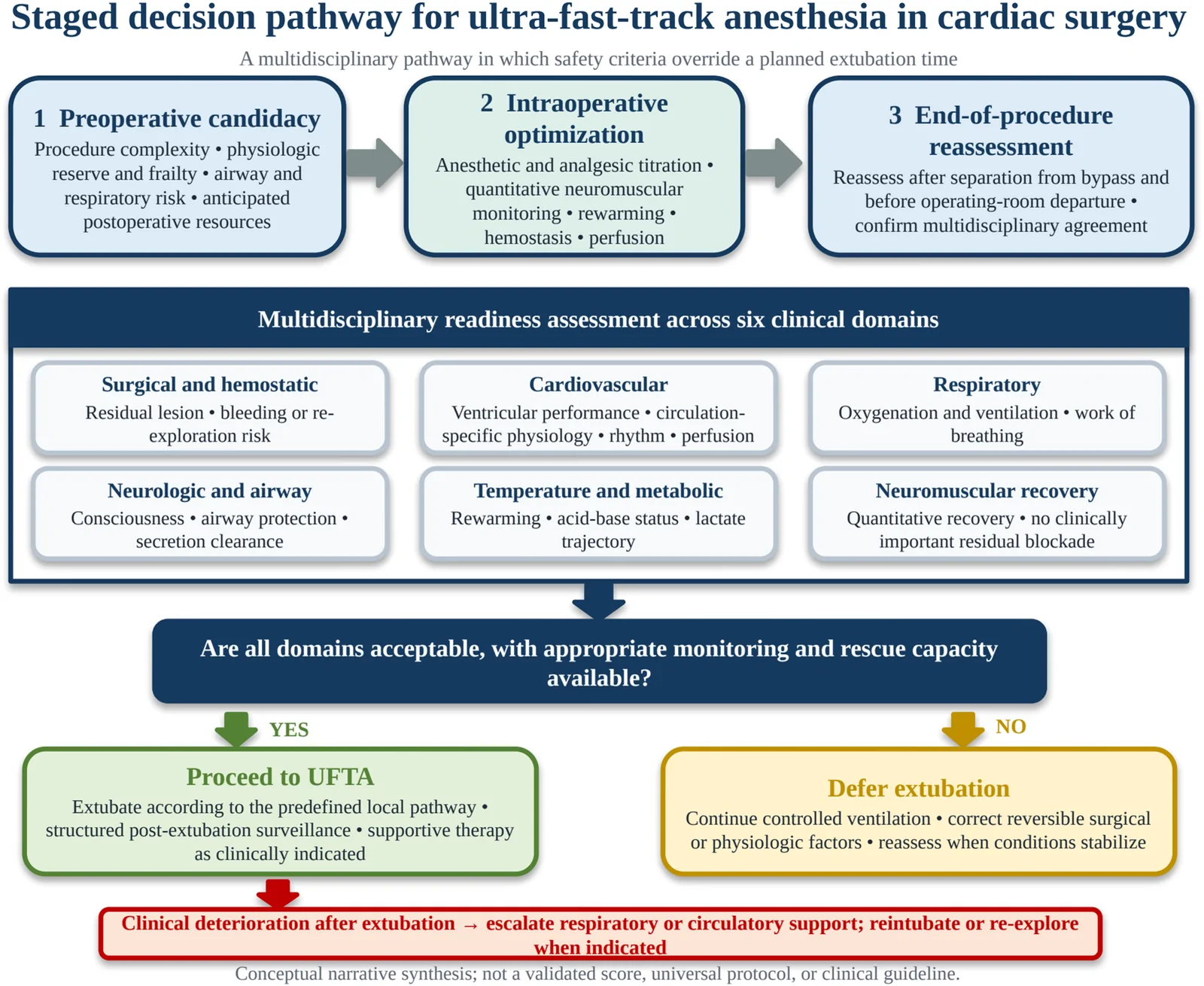
Conceptual staged decision pathway for UFTA in cardiac surgery. Preoperative candidacy is followed by intraoperative optimization and a multidisciplinary end-of-procedure reassessment across surgical/hemostatic, cardiovascular, respiratory, neurologic/airway, temperature/metabolic, and neuromuscular-recovery domains. Cardiovascular assessment includes ventricular performance and circulation-specific physiology rather than assuming a biventricular circulation. If all domains are acceptable and appropriate monitoring and rescue resources are available, the pathway may proceed to extubation with structured post-extubation surveillance; otherwise, ventilation is continued, reversible factors are corrected, and readiness is reassessed. Clinical deterioration after extubation prompts escalation to respiratory or circulatory support and reintubation or re-exploration when indicated. This figure is a narrative synthesis and is not a validated adult or pediatric scoring system, guideline algorithm, or substitute for individualized multidisciplinary judgment. In pediatric single-ventricle pathways, stage-specific physiology supersedes this general framework.

Patient selection for UFTA should be treated as a staged, dynamic decision rather than a single preoperative eligibility judgment. The pathway comprises three linked checkpoints: baseline candidacy before surgery, physiological and surgical reassessment after separation from cardiopulmonary bypass and before operating-room departure, and confirmation that early post-extubation monitoring and rescue resources are appropriate. A favorable profile increases feasibility but does not guarantee extubation success, whereas a new intraoperative or peri-extubation concern should override an initially favorable assessment. Figure 1 presents a general framework for multidisciplinary reassessment rather than a validated adult or pediatric score. In children with single-ventricle circulation, stage-specific physiology supersedes generic ventricular and respiratory criteria.

### Preoperative candidacy

Although UFTA has been associated with shorter ventilation in selected patients, its success relies on strict patient selection and clear separation of three related outcomes: failure to meet early-extubation criteria, reintubation after extubation, and planned delayed extubation. Preoperative candidacy should consider left ventricular function, pulmonary reserve, airway risk, frailty, renal dysfunction, pulmonary hypertension, obesity, and procedure complexity (12, 30, 31). Observational models of delayed extubation can help identify higher-risk patients, but they should not be treated as direct predictors of post-extubation reintubation (30–32). Current evidence is strongest in low- to moderate-risk patients with preserved cardiopulmonary reserve and a realistic postoperative monitoring plan. Key factors warranting caution, individualized assessment, or deferral are summarized in Table 3.

**Table 3:** Phase-specific factors warranting caution, reassessment, or deferral within a dynamic UFTA selection pathway.

Category	Risk factor or relative contraindication	Notes
Preoperative patient factors	Advanced age with frailty or limited physiological reserve	Chronological age alone should not determine eligibility
	Severe left ventricular dysfunction	Assess hemodynamic reserve and anticipated inotropic support
	Severe preoperative pulmonary hypertension	Risk of pulmonary hypertension crisis post-extubation
	Severe obesity, obstructive sleep apnea, or difficult airway	Higher risk of airway obstruction and postoperative respiratory compromise
	Pre-existing renal dysfunction	May affect pharmacokinetics, volume status, acid-base balance, and postoperative recovery
Intraoperative and surgical factors	Prolonged cardiopulmonary bypass or cross-clamp time	May indicate greater physiological stress and delayed recovery
	Redo cardiac surgery	Complex dissection, longer operative time, and higher bleeding or re-exploration risk
	Substantial bleeding or transfusion requirement	May indicate unresolved hemostatic or hemodynamic risk
	Hypothermia at the end of surgery	Affects coagulation and drug metabolism
	Escalating vasoactive or inotropic support after CPB separation	Suggests unstable hemodynamics and should prompt deferral of immediate extubation
	Metabolic acidosis, hyperlactatemia, or inadequate rewarming	Indicates incomplete physiological recovery and warrants reassessment before extubation
	New or worsening ventricular dysfunction on intraoperative TEE	Should prompt reassessment of hemodynamic reserve before extubation
	Residual neuromuscular blockade or inadequate quantitative neuromuscular recovery	Core intraoperative no-go criterion; defer extubation until quantitative recovery is adequate
	Impaired consciousness or inability to protect airway	Core no-go criterion; defer extubation until airway protection and mental status are adequate
	Clinically significant arrhythmia or pacing instability	May compromise immediate post-extubation cardiovascular stability
	Ongoing surgical bleeding or high likelihood of re-exploration	Requires correction before considering immediate extubation
	Major residual lesion or unresolved surgical concern on intraoperative TEE (33)	Potential need for correction or diagnostic clarification outweighs a planned extubation time target
	Pulmonary hypertensive crisis or unstable right-ventricular-pulmonary vascular coupling (37)	Hypoxemia, hypercapnia, acidosis, pain, or loss of controlled ventilation may precipitate deterioration; defer until pulmonary vascular and right-ventricular physiology are stable
	Open sternum, anticipated re-exploration, or ongoing IABP, ECMO, or other temporary mechanical circulatory support (31, 36)	Usually favors planned postoperative ventilation because bleeding, hemodynamic, or rescue requirements remain unresolved
Pediatric and single-ventricle modifiers	Neonatal stage-I palliation or unstable shunt-dependent single-ventricle circulation	Routine immediate extubation is generally inappropriate. Preoperative ventilation, unstable systemic-pulmonary flow balance, hypoxemia or acidosis, residual lesions, pulmonary vascular instability, high or escalating vasoactive support, open sternum, or bleeding should favor planned postoperative ventilation.
Pediatric and single-ventricle modifiers	Stage-II/III cavopulmonary circulation with unfavorable end-of-procedure physiology	The physiologic benefit of spontaneous breathing does not override no-go criteria. Extubation requires acceptable pulmonary vascular and circulation-specific physiology, oxygenation, ventilation, acid-base status, ventricular performance, rhythm, perfusion, hemostasis, low and non-escalating support, airway protection, and immediate rescue capacity.
Pediatric and single-ventricle modifiers	Pediatric procedural complexity expressed by STAT or RACHS-1 category	Use as mortality-risk or case-mix context only. Neither system provides a validated UFTA threshold, and they should not be combined into an unvalidated UFTA score.
Institutional factors	Limited postoperative monitoring or rescue capacity	Institutional resources and staffing are part of candidacy assessment
Peri-extubation and immediate postoperative factors	Persistent hypoxemia, hypercapnia, or excessive work of breathing	Indicates insufficient respiratory reserve and warrants continued ventilatory support or rescue escalation
	Ineffective airway protection, cough, or secretion clearance	Increases the risk of airway obstruction, aspiration, or early respiratory failure
	Uncontrolled pain, residual sedation, or agitation	May impair ventilation, cooperation, and hemodynamic stability
	Hemodynamic instability, evolving low cardiac output, or new rhythm disturbance	Requires stabilization and reassessment before pathway progression

These factors are intended as practical markers for caution, individualized reassessment, or deferral of immediate extubation rather than absolute contraindications. They synthesize evidence on delayed extubation, fast-track protocol failure, frailty assessment, perioperative enhanced-recovery recommendations, and quantitative neuromuscular monitoring (30, 65). Final extubation decisions should be made dynamically after separation from cardiopulmonary bypass and immediately before operating-room departure, taking into account hemodynamic stability, temperature, acid-base status, bleeding risk, airway protection, quantitative neuromuscular recovery, and institutional monitoring and rescue capacity (12, 18).

The factors summarized in Table 3 are not externally validated thresholds or a universal UFTA score. They integrate heterogeneous evidence on delayed extubation, fast-track pathway failure, perioperative readiness, and institutional capability; consequently, they should support multidisciplinary judgment rather than replace it.

Features that may favor consideration of UFTA include elective or lower-complexity surgery, preserved or compensated biventricular function, adequate pulmonary reserve, absence of a predicted difficult airway, limited frailty, and an expectation of uncomplicated hemostasis and recovery. These features should be interpreted as selection markers rather than validated predictors of successful operating-room extubation. Models developed for delayed extubation or fast-track pathway failure address different endpoints and should not be extrapolated directly to post-extubation reintubation or to strict UFTA success.

### Intraoperative go/no-go reassessment

Final extubation decisions should be made after separation from cardiopulmonary bypass and again before leaving the operating room. Bleeding, hypothermia, acidosis, hyperlactatemia, escalating vasoactive requirements, new ventricular dysfunction on TEE, rhythm instability, and re-exploration risk may convert an apparently eligible patient into a poor candidate for immediate extubation (12, 30, 32).

Conversely, conditions supporting progression to immediate extubation include complete rewarming, acceptable gas exchange and acid-base status, stable rhythm, satisfactory ventricular function on TEE, hemostasis without anticipated re-exploration, stable or decreasing vasoactive support, adequate consciousness and airway protection, and quantitative confirmation of neuromuscular recovery. Any discordant finding should trigger deferral rather than pressure to meet a pathway time target.

### Surgical and early postoperative determinants of extubation readiness

Successful early extubation depends on resolution of surgical issues as well as anesthetic recovery. After separation from cardiopulmonary bypass, the surgical and anesthesia teams should use a comprehensive post-repair TEE examination to identify a major residual gradient, clinically important residual regurgitation or shunt, coronary malperfusion, a new regional wall-motion abnormality, worsening ventricular dysfunction, or another unresolved surgical concern (33). A finding that may require immediate correction, additional diagnostic clarification, or observation with controlled ventilation should override a planned UFTA time target. Hemostasis should be evaluated as a trajectory rather than by a universal chest-drain threshold. Persistent surgical bleeding, worsening coagulopathy, ongoing transfusion, concern for tamponade, or a meaningful likelihood of re-exploration favors continued airway control until the source and direction of bleeding are clear (34). Re-exploration after cardiac surgery is associated with substantial morbidity, but current evidence does not validate one bleeding cutoff for immediate extubation across operations and centers.

Ventricular function and perfusion should be interpreted dynamically. A stable preoperative reduction in ejection fraction is not equivalent to new or worsening biventricular dysfunction or postoperative low cardiac output syndrome. Concern increases with difficult separation from bypass, pulmonary edema, impaired peripheral perfusion, rising lactate or persistent acidosis, oliguria, a clinically important rhythm disturbance, or escalating vasoactive-inotropic requirements (35). Stable low-dose support does not by itself establish unsuitability, but an upward support trajectory, evidence of inadequate organ perfusion, or inability to maintain stable hemodynamics should prompt planned ventilation and further treatment. An open sternum or ongoing IABP, ECMO, or other temporary mechanical circulatory support generally indicates unresolved physiologic or rescue needs and therefore favors continued airway control (31). Reports of early extubation in selected LVAD pathways should be treated as experienced-center exceptions rather than evidence that mechanical support is generally compatible with strict UFTA (36).

Pulmonary vascular instability requires separate attention because extubation changes intrathoracic pressure, gas exchange, and sympathetic stimulation. Hypoxemia, hypercapnia, acidosis, hypothermia, pain, or agitation may increase pulmonary vascular resistance and precipitate right-ventricular-pulmonary vascular uncoupling. A recent or incompletely controlled pulmonary hypertensive crisis, worsening right-ventricular dysfunction, unstable oxygenation or pressure, or continuing need for intensive pulmonary vasodilator or ventilatory management should preclude immediate extubation (37). If extubation proceeds after stabilization, it is not the endpoint of pathway assessment: recurrent bleeding, low cardiac output, pulmonary hypertensive deterioration, arrhythmia, and respiratory failure must be actively monitored, with rapid access to noninvasive ventilation, reintubation, surgical re-exploration, and circulatory support.

## Peri-extubation and immediate postoperative suitability

Successful pathway completion also depends on the period immediately surrounding extubation. Persistent hypoxemia or hypercapnia, excessive work of breathing, ineffective cough or secretion clearance, uncontrolled pain, residual sedation, evolving low cardiac output, or new rhythm instability should prompt continued ventilation or early rescue escalation. Even when physiological criteria are met, operating-room extubation may be inappropriate if monitored recovery, experienced staffing, noninvasive respiratory support, and rapid reintubation capability are not reliably available.

## Application of UFTA in adult cardiac surgery

### Current application Status

In adult cardiac surgery, UFTA has expanded at selected centers, but implementation and outcome evidence remain predominantly observational. For example, a six-year single-center retrospective study found that 36.2% of nonemergency isolated CABG patients at that institution were extubated in the operating room (38). A Spanish propensity score-matched analysis compared strict UFTA, defined as intraoperative extubation, with conventional fast-track extubation within 6 h after adult cardiac surgery and reported fewer pulmonary complications without higher reintubation or 30-day mortality; this observational comparison may be affected by patient selection and institutional confounding (8). In addition, a study from the University of Pittsburgh Medical Center (USA) evaluated 652 adult cardiac surgery patients who were extubated within 12 h after surgery, including 165 patients extubated in the operating room. Operating-room extubation was associated with shorter ICU and hospital stays and lower total costs without a significant increase in reintubation; however, these findings arose from a retrospective single-center comparison (39).

Supplementary Table S5 provides a structured comparison of 16 selected studies and evidence syntheses, with separate columns for study design and population, the UFTA or operating-room extubation definition and comparator, ventilation or extubation, ICU LOS, hospital LOS, reintubation, pulmonary complications, mortality, and key limitations. Clinically relevant values unavailable from the accessible report are labeled NR rather than inferred. The studies differ substantially in design, population, intervention, and outcome definitions; they support feasibility in selected settings but do not establish uniform benefits across all cardiac surgical patients (5).

The quantitative evidence summarized in Supplementary Table S5 indicates that the most consistent signal concerns process and resource outcomes: in selected cohorts, UFTA or operating-room extubation is often associated with shorter ventilation, ICU exposure, or hospital stay. Patient-centered safety findings are less consistent. The CARDU-FAST randomized trial did not significantly reduce its primary composite endpoint, while large observational databases have reported associations in opposite directions. Shorter resource use should therefore not be interpreted as proof of causal safety benefit; center experience, dynamic case selection, and heterogeneous outcome definitions remain central to interpretation (40–42).

### Current Application of UFTA in Coronary Artery Bypass Grafting (CABG)

CABG—particularly selected elective cases—represents the most extensively studied adult population for fast-track and ultra-fast-track strategies because procedures are relatively standardized and postoperative hemodynamic or respiratory recovery may be predictable (3, 8). Advances in CPB management, myocardial protection, and monitoring have further enabled UFTA in on-pump CABG when patients are stable after weaning from CPB, adequately rewarmed, and without major bleeding or arrhythmias. In a large propensity score-matched analysis, Carnero-Alcázar et al. reported fewer pulmonary complications with strict UFTA (intraoperative extubation) compared with conventional fast-track extubation within 6 h, without higher reintubation or 30-day mortality (8). In carefully selected patients undergoing minimally invasive cardiac surgery who are extubated on-table and remain hemodynamically stable, postoperative monitoring in an intermediate-care or dedicated recovery unit followed by early ward transfer may be feasible, although current evidence remains observational (7, 43). Therefore, operating-room extubation should not be interpreted as an isolated performance metric without accounting for case mix, surgical complexity, staffing model, and rescue capacity (12, 15).

### Valve surgery

Patients undergoing valve surgery generally exhibit greater heterogeneity in anatomical characteristics, surgical complexity, and comorbidities compared with CABG patients, necessitating a more cautious approach to UFTA in this population. Reports from adjacent procedure groups, including aortic surgery, illustrate that early-extubation feasibility is procedure-specific and should not be extrapolated to elective single-valve or minimally invasive valve pathways without procedure-specific support (32, 44).

For aortic valve replacement (AVR), particularly in patients with degenerative aortic stenosis, very early extubation may be considered in selected patients, provided that cardiopulmonary bypass time is limited, cardiac function recovers adequately after weaning from bypass, and there are no significant conduction disturbances or major bleeding. Kiessling et al. reported that among patients undergoing AVR, the primary predictors of fast-track protocol failure were prolonged bypass duration, extensive intraoperative transfusion, and early postoperative bleeding, although this study did not use the strict within-1-h UFTA definition (32). In mitral valve surgery, minimally invasive techniques (e.g., right mini-thoracotomy or robotic-assisted approaches) may create more favorable conditions for early extubation, but evidence remains procedure- and center-specific (45, 46). Available evidence is concentrated in minimally invasive and elective single-valve procedures, with limited data for combined or complex valve surgery. Bonaros et al. argued from a perspective standpoint that fast-track principles may be incorporated into minimally invasive mitral surgery; this should be interpreted as a pathway concept rather than high-level evidence of safety (47).

### High-complexity cardiac surgery: aortic procedures, reoperations, and circulatory support

Aortic arch procedures and operations requiring hypothermic circulatory arrest have traditionally prompted planned postoperative ventilation because prolonged CPB, systemic inflammation, coagulopathy, incomplete rewarming, and neurologic uncertainty can delay safe extubation (48). Recent experienced-center evidence challenges procedure-based exclusion from ORE. In a single-center descriptive cohort of 265 consecutive aortic operations, Salna et al. reported operating-room extubation in 244 patients (92%), including 164 of 180 patients (91.1%) who underwent moderate or deep hypothermic circulatory arrest. Operating-room extubation was associated with earlier mobilization and shorter ICU and hospital stays, with low rates of reintubation and major morbidity. However, the nonrandomized single-center design, strong institutional pathway, and small ICU-extubation comparison group limit causal inference and generalizability (44). Carnero-Alcázar et al. included selected ascending-aortic procedures within a mixed cardiac-surgery cohort and found no overall increase in complications with UFTA (8). Accordingly, circulatory arrest, reoperation, or procedural complexity should not be treated as automatic exclusions; nevertheless, ORE after arch or complex aortic surgery should remain contingent on experienced-center protocols and unequivocal neurologic, thermal, hemostatic, and hemodynamic recovery. Evidence remains particularly limited for emergency acute aortic dissection (49). Selected minimally invasive HeartMate 3 programs also indicate that durable LVAD implantation does not automatically preclude very early extubation, but these reports remain investigational (50).

### Heart transplantation and durable ventricular assist devices

Emerging reports extend ORE/UFTA to carefully selected advanced heart-failure procedures. In a retrospective single-center study of 53 adult LVAD implantations, Zayat et al. reported successful UFTA in 13 selected INTERMACS 3–4 patients; after propensity matching, UFTA was associated with lower incidences of pneumonia, delirium, and right-ventricular failure and with a shorter ICU stay, whereas four-year survival did not differ (36). Their pathway allowed extubation in the operating room or within 4 h after surgery, so it does not represent strict UFTA in every patient, and the small as-treated cohort remains susceptible to selection and residual confounding. In pediatric orthotopic heart transplantation, Schnittman et al. described four uncomplicated cases extubated in the operating room; mild respiratory acidosis and hypercapnia resolved within 6–12 h, and no patient required reintubation or died (51). These reports support feasibility in highly selected programs rather than routine application or proven causal benefit (50). Direct evidence for pediatric VAD implantation remains insufficient to justify extrapolation from adult LVAD cohorts.

## Why procedural complexity modifies UFTA suitability

Procedure type should be interpreted as a proxy for anticipated physiologic and rescue burden rather than as a stand-alone eligibility rule. Selected elective isolated CABG and uncomplicated isolated valve or minimally invasive procedures may be more compatible with UFTA when operative duration and surgical trauma are predictable, CPB and cross-clamp exposure are limited, hemostasis is secure, and ventricular function and rhythm recover promptly (38, 45). These features permit respiratory, neurologic, and hemodynamic readiness to be assessed before operating-room departure; however, the reported advantages remain susceptible to case selection and center effects.

Conversely, redo sternotomy may require extensive adhesiolysis and increase the risk of injury during re-entry, transfusion, prolonged operative time, or re-exploration (52). Combined procedures and prolonged CPB or cross-clamp exposure may compound hypothermia, inflammation, coagulopathy, vasoplegia, myocardial dysfunction, and low cardiac output, lowering the threshold for planned postoperative ventilation (32). Complex aortic procedures involving circulatory arrest add neurologic, thermal, and hemostatic uncertainty (44, 49). The need for an intra-aortic balloon pump, extracorporeal membrane oxygenation, escalating or high-dose vasoactive support, or unresolved ventricular failure indicates that continued airway control and intensive monitoring may be preferable (31). These contexts are not absolute contraindications: uncomplicated re-entry and highly selected mechanical-support cohorts have been extubated early, but the final go/no-go decision should be based on physiology and rescue requirements at the end of surgery rather than the planned procedure alone.

## UFTA in special populations

### Pediatric cardiac surgery

In pediatric cardiac surgery, early extubation predates its widespread use in adults because prolonged ventilation and deep sedation—especially in infants—are linked to more respiratory complications, delayed enteral feeding, and longer ICU stays. Accordingly, some pediatric centers routinely use fast-track pathways, including intraoperative extubation (53). Multicenter observational data describe on-table extubation across corrective and palliative congenital procedures and identify higher-risk patterns in palliative and shunt-dependent physiology (54). A 2026 systematic review of comparative studies found that on-table extubation was generally associated with shorter ICU and hospital stay, whereas reintubation findings were inconsistent and no difference in perioperative mortality was identified (55). The marked heterogeneity and selection bias across studies preclude causal or equivalence conclusions (55). Neonates and children undergoing high-complexity or single-ventricle procedures require separate consideration rather than automatic exclusion. In a neonatal cohort, immediate extubation was achieved in selected patients, while higher gestational age, absence of preoperative ventilatory support, and shorter cardiopulmonary bypass time were associated with success (56).

### Age and developmental considerations

Age modifies, but does not independently determine, extubation readiness. Neonates should be considered separately from older infants and children because developmental physiology and the prevalence of preoperative ventilatory support differ substantially across these groups. Immediate extubation is feasible in selected neonates, but the probability of success is lower when gestational age is low, preoperative ventilatory support is required, or cardiopulmonary bypass is prolonged (56). Older infants and children undergoing lower-complexity corrective procedures, with adequate weight, no preoperative ventilation, and stable end-of-procedure physiology, are generally more favorable candidates (57). Evidence using extubation within 24 h should be described as early extubation rather than strict UFTA or operating-room extubation.

### Procedure complexity and risk classification

Procedure complexity should inform, but not replace, perioperative assessment. The Society of Thoracic Surgeons-European Association for Cardio-Thoracic Surgery (STAT) mortality categories and the Risk Adjustment for Congenital Heart Surgery (RACHS-1) system were developed to stratify procedure-related mortality or case mix; neither is a validated model for strict UFTA success, operating-room extubation, or reintubation (58, 59). Higher categories can identify populations in whom caution is warranted, but no STAT or RACHS-1 threshold should be treated as an automatic indication or contraindication (59). In observational cohorts, lower RACHS-1 category, older age, greater weight, absence of preoperative pulmonary hypertension, shorter bypass and cross-clamp times, and lower vasoactive-inotropic requirements were associated with extubation within 24 h (60). These factors are therefore best interpreted as selection markers whose relevance must be reassessed at the end of surgery. Accordingly, STAT and RACHS-1 should not be combined into an unvalidated pediatric UFTA score.

### Single-ventricle physiology, pulmonary hypertension, and dynamic selection

Single-ventricle physiology is stage-specific and should not be treated as a uniform indication for or against UFTA. After Norwood or other stage-I palliation for hypoplastic left heart syndrome and shunt-dependent circulation, the need to balance systemic and pulmonary blood flow, frequent low weight or preoperative ventilation, and the risk of postoperative hemodynamic or airway instability make routine immediate extubation inappropriate (54). By contrast, selected patients after superior cavopulmonary connection (Glenn or stage II) or Fontan completion may tolerate early or operating-room extubation when oxygenation, ventilation, rhythm, perfusion, and vasoactive requirements are favorable (61–63). Spontaneous negative intrathoracic pressure provides a physiologic rationale for avoiding unnecessary positive-pressure ventilation in cavopulmonary circulations, but current clinical evidence is observational and does not establish that early extubation is mandatory or causally improves outcomes.

For stage-II/III cavopulmonary pathways, progression toward extubation should require acceptable pulmonary vascular and circulation-specific physiology, stable oxygenation and ventilation, satisfactory acid-base status, preserved ventricular performance, stable rhythm and perfusion, secure hemostasis, low and non-escalating vasoactive-inotropic support, adequate airway protection, and immediate rescue capacity. Spontaneous negative intrathoracic pressure can support passive cavopulmonary blood flow, giving liberation from unnecessary positive-pressure ventilation a stronger physiologic rationale than in most biventricular pathways; this rationale does not make early or operating-room extubation mandatory. Hypoxemia, hypercapnia or acidosis, elevated pulmonary vascular resistance, ventricular dysfunction, significant arrhythmia, a major residual lesion, open sternum or active bleeding, or escalating support should prompt planned postoperative ventilation. These factors are stage-specific decision modifiers, not a validated pediatric UFTA score (64).

Pulmonary hypertension is an important warning signal rather than an absolute contraindication. Uncontrolled pulmonary vascular reactivity, hypoxemia, hypercarbia or acidosis, right-ventricular dysfunction, escalating vasoactive support, a pulmonary hypertensive crisis, major residual lesions, open sternum, active bleeding, or inadequate airway protection should prompt planned postoperative ventilation (60). Favorable pediatric candidates should demonstrate stable oxygenation and ventilation, acceptable pulmonary pressures and ventricular performance, secure hemostasis, low and non-escalating vasoactive-inotropic support, normothermia, adequate analgesia and neuromuscular recovery, and immediate access to monitored post-extubation support and reintubation (37). These criteria are dynamic decision markers, not a validated pediatric UFTA score.

### Older and frail patients

Traditionally, advanced age was seen as a reason for caution with UFTA because frailty, reduced physiological reserve, impaired baseline function or cognition, limited pulmonary reserve, and comorbidity burden are common in older adults. However, recent evidence has begun to challenge chronological age as a standalone exclusion criterion. Frailty assessment may be more informative than chronological age alone, although no frailty-specific UFTA threshold has been validated (65). In a study by Parody Cuerda et al. involving 1,498 cardiac surgery patients managed with a standardized UFTA protocol, high rates of successful intraoperative extubation were observed even among older adults when perioperative care was optimized (31). Notably, the primary determinants of ultra-fast extubation failure in that study were the need for preoperative mechanical circulatory support (such as an intra-aortic balloon pump), the requirement for high-dose vasoactive agents intraoperatively or postoperatively, and the complexity or urgency of the surgical procedure—rather than chronological age. Prolonged mechanical ventilation and deep sedation in older adults are associated with delirium, ICU-acquired weakness, and delayed functional recovery. Earlier extubation may reduce sedative exposure and facilitate mobilization, but any association with lower delirium should be interpreted cautiously because causality has not been established.

### Patients with low cardiac function

Patients with a significantly reduced ejection fraction or advanced heart failure have traditionally been viewed as poor candidates for fast-track anesthesia or UFTA, primarily due to concerns regarding postoperative hemodynamic instability. However, with ongoing advances in anesthetic pharmacology and perioperative monitoring, this paradigm is gradually evolving.

Evidence for UFTA in patients with markedly reduced ejection fraction (<30%) remains limited and should be treated primarily as an evidence gap. Data remain sparse, and in selected low-ejection-fraction cases, early extubation should be considered only in exceptional, highly selected cases within experienced programs when hemodynamic stability is maintained intraoperatively and after separation from cardiopulmonary bypass (50). Nevertheless, applying UFTA in patients with severely impaired cardiac function requires highly individualized decision-making. If high-dose vasopressor support, pulmonary edema, metabolic acidosis, or inadequate perfusion is present, extubation should be deferred.

We performed a comparative review of multiple studies to evaluate the suitability of UFTA across different types of cardiac surgery and to identify clinical scenarios in which UFTA is not recommended. These findings are summarized in Supplementary Table S6.

## Challenges facing UFTA implementation

### Multidisciplinary coordination

Successful implementation of UFTA depends on addressing several challenges, chief among which are stringent patient selection and highly coordinated perioperative management. Close collaboration within a multidisciplinary team is essential: anesthesiology, surgical, and ICU teams all need to be aligned in both principles and practice (7, 12). Anesthesiologists must be proficient in the use of short-acting anesthetic agents and capable of dynamically adjusting anesthetic depth in response to the surgical progress. Surgeons, in turn, should minimize any unnecessary intraoperative delays so that by the end of the procedure the patient's physiological status is optimized. Prior to extubation, it is critical to confirm that all surgery-related issues—such as achieving adequate hemostasis and stable cardiac function—have been fully addressed. The ICU team must also be prepared to care for patients extubated at the end of surgery, which entails enhanced early postoperative monitoring and the availability of appropriate support resources (e.g., noninvasive ventilation, high-flow nasal oxygen therapy, and structured postoperative analgesia protocols) (7, 12).

### Institutional readiness and rescue capacity

Institutional readiness is equally important (66). UFTA should be implemented as a pathway with predefined failure criteria and rescue protocols rather than as an individual clinician-dependent decision. Programs should specify reintubation triggers, difficult-airway backup, availability of HFNC or noninvasive ventilation, criteria for ICU or step-down transfer, and staffing models that remain reliable during nights and weekends (12, 15).

## Contingency planning and extubation failure response

Another major challenge is contingency planning and risk management. If any signs of respiratory compromise occur after extubation, the clinical team must respond rapidly with appropriate interventions. This necessitates that all team members have a thorough understanding of the risks associated with UFTA and receive proper training, including simulation-based drills for managing extubation failure. Before proceeding with extubation in a UFTA case, the anesthesiologist should rigorously reassess that all extubation criteria have been fully met. Objective elements such as quantitative neuromuscular monitoring, arterial blood gas analysis, temperature, hemodynamic stability, and airway-protection assessment may be incorporated into local readiness criteria to minimize the risk of extubation failure (19, 30, 50). Local UFTA programs should prospectively audit extubation failure and reintubation events.

## Limitations of the current evidence

This narrative review has several limitations. At the level of the underlying evidence, definitions of UFTA, operating-room extubation, and fast-track extubation vary across studies, creating definition-dependent misclassification and limiting direct comparison (8). Most available evidence derives from single-center retrospective cohorts and is susceptible to confounding by indication, publication bias, center-expertise effects, and preferential selection of lower-risk patients and less complex procedures for immediate extubation (5). Outcome definitions, postoperative care pathways, and discharge criteria also differ substantially across institutions, and there is no widely adopted core outcome set for UFTA research. At the level of this review, the literature search and study selection were narrative rather than systematic: no protocol was registered, duplicate independent screening was not performed, and no formal risk-of-bias or certainty-of-evidence assessment was undertaken. Evidence from adult, pediatric, minimally invasive, and conventional sternotomy populations should therefore not be considered interchangeable, and long-term patient-centered outcomes remain insufficiently characterized (18). The English-language restriction, non-duplicate narrative selection process, and reliance on heterogeneous direct and indirect evidence may have introduced language and selection bias.

## Discussion and future perspectives

Procedure labels should therefore function as prompts for dynamic reassessment rather than determine extubation: bleeding, temperature, neurologic recovery, ventricular performance and circulation-specific physiology, acid-base status, and the need for circulatory support at the end of surgery remain decisive (12).

UFTA has emerged as a selective component of enhanced recovery after cardiac surgery. It is consistently associated with shorter invasive ventilation in appropriately selected patients, but current evidence does not establish that operating-room extubation universally reduces reintubation, mortality, or other major outcomes (8).

Nevertheless, UFTA is not suitable for all patients. Future work should standardize UFTA definitions and core outcome sets, distinguish procedure- and physiology-specific populations, including pediatric single-ventricle pathways, and prospectively evaluate whether early extubation improves outcomes beyond shorter ventilation (5, 12). Large multicenter studies should clarify which patients benefit most, particularly frail older adults, patients with impaired ventricular function, and those undergoing highly complex procedures such as DHCA or prolonged CPB. Patient-selection models should incorporate dynamic intraoperative variables, including vasoactive support, lactate or acidosis, TEE findings, rhythm instability, bleeding, and re-exploration risk. Future implementation studies should also test structured extubation-readiness tools, no-go criteria, rescue escalation pathways, and post-extubation support strategies rather than treating operating-room extubation as an isolated endpoint.

## Conclusion

UFTA represents a selective, multidisciplinary enhanced-recovery strategy for cardiac surgery. In appropriately selected patients and within institutions with standardized readiness criteria, postoperative monitoring, and rescue capacity, UFTA has been associated with shorter invasive ventilation and may improve recovery and resource-use outcomes in selected settings (8). Future studies should standardize definitions and core outcomes, refine procedure-specific patient selection, incorporate dynamic intraoperative decision points, and determine whether benefits extend beyond shorter ventilation without compromising safety.
