# Supplementary material for: Ultra-fast-track anesthesia in cardiac surgery: perioperative outcomes, patient selection, and implementation strategies—a narrative review
Source: Front Cardiovasc Med. 2026 Aug 12;13:1914025. doi: 10.3389/fcvm.2026.1914025 (PMC13507958; doi:10.3389/fcvm.2026.1914025)
Supplement: Supplementary file 1 [file Datasheet1.docx]

**Supplementary Methods: Search Strategies and Study-Selection Record**

**Supplementary Figure S1. PRISMA-style flow diagram of literature identification, title-and-abstract screening, focused full-text verification, and narrative citation reconciliation. The diagram presents the reconstructed workflow for this narrative review and is not a formal systematic-review inclusion cascade.**

For rapid review, the flow diagram and compact accounting tables are presented before the full search strings. Complete database-specific strategies are retained in Supplementary Appendix A.

**Supplementary Table S1. Databases, coverage, and retrieval**

| **Database / platform** | **Search date** | **Coverage** | **Overlapping domain hits** | **Within-database unique** |
| --- | --- | --- | --- | --- |
| PubMed (NCBI; includes MEDLINE-indexed records) | 21 Jul 2026 | Inception-search date; English limit | 2,063 | 1,431 |
| Embase (Embase.com) | 22 Jul 2026 | Inception-search date; English limit | 4,230 | 2,821 |
| Web of Science Core Collection (Clarivate) | 22 Jul 2026 | Inception-search date; English limit | 2,882 | 1,978 |
| CINAHL (EBSCOhost) | 22 Jul 2026 | Inception-search date; English limit | 469 | 350 |
| Cochrane Library (CDSR and CENTRAL) | 22 Jul 2026 | Inception-search date; English eligibility at screening | 1,764 | 1,323 |
| Total |  |  | 11,408 | 7,903 |

**Supplementary Table S2. Search domains**

| **Domain** | **Scope** |
| --- | --- |
| 1. Direct UFTA/extubation | UFTA; operating-room, on-table, intraoperative, immediate, or early extubation |
| 2. Anesthetic techniques | Anesthetic management; TIVA; volatile/balanced anesthesia; short-acting, opioid-sparing, opioid-free, and multimodal regimens |
| 3. Neuromuscular recovery | Quantitative monitoring; train-of-four; residual blockade; sugammadex, neostigmine, and reversal strategies |
| 4. ERAS/pathways | Enhanced recovery; fast-track pathways; extubation protocols; implementation |
| 5. Regional analgesia | Erector spinae, paravertebral, parasternal, transversus thoracis, serratus, intercostal, and related techniques |
| 6. Respiratory support | HFNC/HFNO; NIV; CPAP; BiPAP; post-extubation support and rescue |
| 7. Selection/failure | Readiness; delayed extubation; failure; predictors; risk factors; feasibility; adherence; center experience |

**Eligibility, selection, and evidence directness**

Eligible sources comprised randomized and nonrandomized clinical studies, systematic reviews, meta-analyses, and professional guidance involving adult or pediatric cardiac surgery. Eligible topics included strict UFTA (operating-room or within-1-hour extubation), 1-6-hour fast-track extubation as contextual evidence, relevant perioperative pathways or techniques, and patient selection, readiness, failure, rescue, or implementation. Smaller observational studies and case series were considered for emerging techniques, uncommon procedures, or special populations.

We excluded noncardiac surgery, peripheral vascular or abdominal aortic surgery, transcatheter-only and nonsurgical critical-care populations without separable cardiac-surgical data, nonclinical studies, duplicate or inferior reports, nondata records, and studies without relevant extubation, ventilation, respiratory-safety, ICU-use, recovery, selection, or implementation information. Evidence was classified as direct strict-UFTA evidence, indirect 1-6-hour fast-track evidence, or indirect pathway-component evidence. Pathway-component evidence was not interpreted as establishing that any individual intervention independently improved UFTA success. Sources outside the clinical eligibility criteria could be retained only for clearly identified background, mechanistic, or professional-guidance context and were not used as direct evidence for cardiac-surgical outcomes.

**Supplementary Table S3. Selection accounting**

| **Stage** | **Records** |
| --- | --- |
| Overlapping records retrieved across seven search domains | 11,408 |
| Within-database unions supplied to cross-database deduplication | 7,903 |
| Candidate records after identifier-based deduplication | 5,075 |
| Exact normalized-title duplicates adjudicated | 730 |
| Report-level records screened by title/abstract | 4,345 |
| Excluded after title/abstract screening | 1,426 |
| Broad potentially relevant evidence pool | 2,919 |
| Focused author-confirmed full-text verification subset (PubMed-prioritized records) | 117 |
| Final manuscript unique-source set | 73 |
| Exact-title-linked citations meeting clinical eligibility criteria | 45 |
| Exact-title-linked citation retained only as indirect pharmacological context | 1 |
| Remaining citations manually verified by the authors | 27 |
| Focused author-audit records reviewed and confirmed | 27 / 27 |

The 2,919 potentially relevant records represent a broad title-and-abstract evidence pool rather than an exhaustively assessed systematic-review inclusion set. Sources for the narrative synthesis were purposively selected according to relevance to the review questions, evidence directness, study design, completeness of outcome reporting, coverage of the predefined domains, and avoidance of redundant reports.

**Supplementary Table S4. Title/abstract exclusion reasons**

| **Reason** | **Records** |
| --- | --- |
| Noncardiac surgical population | 228 |
| Transcatheter-only or nonsurgical population | 186 |
| No relevant extubation, ventilation, respiratory-safety, ICU/recovery, or implementation outcome | 447 |
| Nonclinical record | 16 |
| Protocol, registration, correction, or other nondata record | 429 |
| Duplicate or inferior report | 23 |
| Other out-of-scope reason | 97 |

**Screening roles and limitations**

S.Z., J.Y., and H.D. performed the original searches and initial title-and-abstract screening. X.W., M.Cui, M.Chen, J.M., and Y.L. reviewed the original evidence set, and disagreements were resolved through discussion. During revision, computer-assisted reconstruction, deduplication, and preliminary classification generated a focused audit set containing flagged and citation-linked records. S.Z. reviewed all 27 focused audit records and confirmed the aggregate selection outputs. The authors also confirmed full-text verification of the 117-record PubMed-prioritized subset used for focused source validation. This focused audit did not constitute duplicate independent screening. The English-language restriction and narrative selection process may have introduced language and selection bias.

Of the final manuscript's 73 unique sources, 46 were linked by exact title to the reconstructed five-database set. Forty-five met the clinical eligibility criteria. One linked MitraClip report fell under X2 (transcatheter-only or nonsurgical) and was retained solely as indirect pharmacological context, not as direct evidence for cardiac-surgical outcomes. The remaining 27 non-linked sources were manually verified by the authors. These figures describe citation reconciliation for a narrative review rather than an exhaustive systematic-review full-text inclusion process.

Supplementary Appendix A. Full database-specific search strategies

**S1. PubMed/MEDLINE**

**01_direct_ufta_extubation**

("Cardiac Surgical Procedures"[Mesh] OR "cardiac surgery"[tiab] OR "cardiac surgical"[tiab] OR "heart surgery"[tiab] OR "open heart surgery"[tiab] OR "open-heart surgery"[tiab] OR "coronary artery bypass"[tiab] OR CABG[tiab] OR "valve surgery"[tiab] OR "off-pump coronary artery bypass"[tiab] OR OPCAB[tiab] OR "minimally invasive cardiac surgery"[tiab] OR "robotic cardiac surgery"[tiab] OR "aortic valve replacement"[tiab] OR "mitral valve surgery"[tiab] OR "thoracic aortic surgery"[tiab] OR "aortic root surgery"[tiab] OR "ascending aortic surgery"[tiab] OR "aortic arch surgery"[tiab] OR hemiarch[tiab] OR "type A aortic dissection"[tiab] OR "congenital heart surgery"[tiab] OR "congenital heart disease"[tiab] OR "ventricular septal defect"[tiab] OR "atrial septal defect"[tiab] OR "adult congenital heart disease"[tiab] OR "pediatric cardiac surgery"[tiab] OR "paediatric cardiac surgery"[tiab] OR "neonatal cardiac surgery"[tiab] OR "infant cardiac surgery"[tiab] OR "heart transplantation"[tiab] OR "cardiac transplantation"[tiab] OR "ventricular assist device"[tiab] OR LVAD[tiab]) AND ("ultra-fast-track"[tiab] OR "ultra fast-track"[tiab] OR "ultra fast track"[tiab] OR "fast-track cardiac anesthesia"[tiab] OR "fast-track cardiac anaesthesia"[tiab] OR "fast-track extubation"[tiab] OR "fast track extubation"[tiab] OR "operating room extubation"[tiab] OR "operating-room extubation"[tiab] OR "on-table extubation"[tiab] OR "on table extubation"[tiab] OR "intraoperative extubation"[tiab] OR "immediate extubation"[tiab] OR "early extubation"[tiab] OR "early tracheal extubation"[tiab]) AND english[Language] AND ("1800/01/01"[Date - Publication] : "2026/07/21"[Date - Publication])

**02_anesthetic_techniques**

("Cardiac Surgical Procedures"[Mesh] OR "cardiac surgery"[tiab] OR "cardiac surgical"[tiab] OR "heart surgery"[tiab] OR "open heart surgery"[tiab] OR "open-heart surgery"[tiab] OR "coronary artery bypass"[tiab] OR CABG[tiab] OR "valve surgery"[tiab] OR "off-pump coronary artery bypass"[tiab] OR OPCAB[tiab] OR "minimally invasive cardiac surgery"[tiab] OR "robotic cardiac surgery"[tiab] OR "aortic valve replacement"[tiab] OR "mitral valve surgery"[tiab] OR "thoracic aortic surgery"[tiab] OR "aortic root surgery"[tiab] OR "ascending aortic surgery"[tiab] OR "aortic arch surgery"[tiab] OR hemiarch[tiab] OR "type A aortic dissection"[tiab] OR "congenital heart surgery"[tiab] OR "congenital heart disease"[tiab] OR "ventricular septal defect"[tiab] OR "atrial septal defect"[tiab] OR "adult congenital heart disease"[tiab] OR "pediatric cardiac surgery"[tiab] OR "paediatric cardiac surgery"[tiab] OR "neonatal cardiac surgery"[tiab] OR "infant cardiac surgery"[tiab] OR "heart transplantation"[tiab] OR "cardiac transplantation"[tiab] OR "ventricular assist device"[tiab] OR LVAD[tiab]) AND ("anesthetic technique"[tiab] OR "anaesthetic technique"[tiab] OR "anesthetic agent"[tiab] OR "anaesthetic agent"[tiab] OR "total intravenous anesthesia"[tiab] OR "total intravenous anaesthesia"[tiab] OR TIVA[tiab] OR "volatile anesthesia"[tiab] OR "volatile anaesthesia"[tiab] OR "opioid-sparing"[tiab] OR "opioid sparing"[tiab] OR "multimodal analgesia"[tiab]) AND (extubat*[tiab] OR "ventilation duration"[tiab] OR "mechanical ventilation time"[tiab]) AND english[Language] AND ("1800/01/01"[Date - Publication] : "2026/07/21"[Date - Publication])

**03_neuromuscular_monitoring_reversal**

("Cardiac Surgical Procedures"[Mesh] OR "cardiac surgery"[tiab] OR "cardiac surgical"[tiab] OR "heart surgery"[tiab] OR "open heart surgery"[tiab] OR "open-heart surgery"[tiab] OR "coronary artery bypass"[tiab] OR CABG[tiab] OR "valve surgery"[tiab] OR "off-pump coronary artery bypass"[tiab] OR OPCAB[tiab] OR "minimally invasive cardiac surgery"[tiab] OR "robotic cardiac surgery"[tiab] OR "aortic valve replacement"[tiab] OR "mitral valve surgery"[tiab] OR "thoracic aortic surgery"[tiab] OR "aortic root surgery"[tiab] OR "ascending aortic surgery"[tiab] OR "aortic arch surgery"[tiab] OR hemiarch[tiab] OR "type A aortic dissection"[tiab] OR "congenital heart surgery"[tiab] OR "congenital heart disease"[tiab] OR "ventricular septal defect"[tiab] OR "atrial septal defect"[tiab] OR "adult congenital heart disease"[tiab] OR "pediatric cardiac surgery"[tiab] OR "paediatric cardiac surgery"[tiab] OR "neonatal cardiac surgery"[tiab] OR "infant cardiac surgery"[tiab] OR "heart transplantation"[tiab] OR "cardiac transplantation"[tiab] OR "ventricular assist device"[tiab] OR LVAD[tiab]) AND ("Neuromuscular Monitoring"[Mesh] OR "neuromuscular monitoring"[tiab] OR "neuromuscular blockade"[tiab] OR "neuromuscular reversal"[tiab] OR "residual neuromuscular blockade"[tiab] OR "train-of-four"[tiab] OR "train of four"[tiab] OR sugammadex[tiab] OR neostigmine[tiab]) AND (extubat*[tiab] OR respiratory[tiab] OR "mechanical ventilation"[tiab]) AND english[Language] AND ("1800/01/01"[Date - Publication] : "2026/07/21"[Date - Publication])

**04_eras_pathways_protocols**

("Cardiac Surgical Procedures"[Mesh] OR "cardiac surgery"[tiab] OR "cardiac surgical"[tiab] OR "heart surgery"[tiab] OR "open heart surgery"[tiab] OR "open-heart surgery"[tiab] OR "coronary artery bypass"[tiab] OR CABG[tiab] OR "valve surgery"[tiab] OR "off-pump coronary artery bypass"[tiab] OR OPCAB[tiab] OR "minimally invasive cardiac surgery"[tiab] OR "robotic cardiac surgery"[tiab] OR "aortic valve replacement"[tiab] OR "mitral valve surgery"[tiab] OR "thoracic aortic surgery"[tiab] OR "aortic root surgery"[tiab] OR "ascending aortic surgery"[tiab] OR "aortic arch surgery"[tiab] OR hemiarch[tiab] OR "type A aortic dissection"[tiab] OR "congenital heart surgery"[tiab] OR "congenital heart disease"[tiab] OR "ventricular septal defect"[tiab] OR "atrial septal defect"[tiab] OR "adult congenital heart disease"[tiab] OR "pediatric cardiac surgery"[tiab] OR "paediatric cardiac surgery"[tiab] OR "neonatal cardiac surgery"[tiab] OR "infant cardiac surgery"[tiab] OR "heart transplantation"[tiab] OR "cardiac transplantation"[tiab] OR "ventricular assist device"[tiab] OR LVAD[tiab]) AND ("Enhanced Recovery After Surgery"[Mesh] OR ERAS[tiab] OR "enhanced recovery"[tiab] OR "perioperative pathway"[tiab] OR "clinical pathway"[tiab] OR "fast-track protocol"[tiab] OR "fast track protocol"[tiab] OR "extubation protocol"[tiab]) AND (extubat*[tiab] OR "mechanical ventilation"[tiab] OR "ventilation duration"[tiab]) AND english[Language] AND ("1800/01/01"[Date - Publication] : "2026/07/21"[Date - Publication])

**05_regional_analgesia**

("Cardiac Surgical Procedures"[Mesh] OR "cardiac surgery"[tiab] OR "cardiac surgical"[tiab] OR "heart surgery"[tiab] OR "open heart surgery"[tiab] OR "open-heart surgery"[tiab] OR "coronary artery bypass"[tiab] OR CABG[tiab] OR "valve surgery"[tiab] OR "off-pump coronary artery bypass"[tiab] OR OPCAB[tiab] OR "minimally invasive cardiac surgery"[tiab] OR "robotic cardiac surgery"[tiab] OR "aortic valve replacement"[tiab] OR "mitral valve surgery"[tiab] OR "thoracic aortic surgery"[tiab] OR "aortic root surgery"[tiab] OR "ascending aortic surgery"[tiab] OR "aortic arch surgery"[tiab] OR hemiarch[tiab] OR "type A aortic dissection"[tiab] OR "congenital heart surgery"[tiab] OR "congenital heart disease"[tiab] OR "ventricular septal defect"[tiab] OR "atrial septal defect"[tiab] OR "adult congenital heart disease"[tiab] OR "pediatric cardiac surgery"[tiab] OR "paediatric cardiac surgery"[tiab] OR "neonatal cardiac surgery"[tiab] OR "infant cardiac surgery"[tiab] OR "heart transplantation"[tiab] OR "cardiac transplantation"[tiab] OR "ventricular assist device"[tiab] OR LVAD[tiab]) AND ("regional anesthesia"[tiab] OR "regional anaesthesia"[tiab] OR "regional analgesia"[tiab] OR "fascial plane block"[tiab] OR "erector spinae plane block"[tiab] OR "paravertebral block"[tiab] OR "parasternal block"[tiab] OR "serratus anterior plane block"[tiab] OR "intercostal nerve block"[tiab]) AND (extubat*[tiab] OR "ventilation duration"[tiab] OR "mechanical ventilation time"[tiab] OR "opioid consumption"[tiab] OR "enhanced recovery"[tiab]) AND english[Language] AND ("1800/01/01"[Date - Publication] : "2026/07/21"[Date - Publication])

**06_postextubation_respiratory_support**

("Cardiac Surgical Procedures"[Mesh] OR "cardiac surgery"[tiab] OR "cardiac surgical"[tiab] OR "heart surgery"[tiab] OR "open heart surgery"[tiab] OR "open-heart surgery"[tiab] OR "coronary artery bypass"[tiab] OR CABG[tiab] OR "valve surgery"[tiab] OR "off-pump coronary artery bypass"[tiab] OR OPCAB[tiab] OR "minimally invasive cardiac surgery"[tiab] OR "robotic cardiac surgery"[tiab] OR "aortic valve replacement"[tiab] OR "mitral valve surgery"[tiab] OR "thoracic aortic surgery"[tiab] OR "aortic root surgery"[tiab] OR "ascending aortic surgery"[tiab] OR "aortic arch surgery"[tiab] OR hemiarch[tiab] OR "type A aortic dissection"[tiab] OR "congenital heart surgery"[tiab] OR "congenital heart disease"[tiab] OR "ventricular septal defect"[tiab] OR "atrial septal defect"[tiab] OR "adult congenital heart disease"[tiab] OR "pediatric cardiac surgery"[tiab] OR "paediatric cardiac surgery"[tiab] OR "neonatal cardiac surgery"[tiab] OR "infant cardiac surgery"[tiab] OR "heart transplantation"[tiab] OR "cardiac transplantation"[tiab] OR "ventricular assist device"[tiab] OR LVAD[tiab]) AND ("high-flow nasal cannula"[tiab] OR "high flow nasal cannula"[tiab] OR "high-flow nasal oxygen"[tiab] OR "high flow nasal oxygen"[tiab] OR HFNC[tiab] OR HFNO[tiab] OR "Noninvasive Ventilation"[Mesh] OR "noninvasive ventilation"[tiab] OR "non-invasive ventilation"[tiab] OR NIV[tiab] OR CPAP[tiab] OR BiPAP[tiab]) AND (postextubat*[tiab] OR post-extubat*[tiab] OR extubat*[tiab] OR reintubat*[tiab]) AND english[Language] AND ("1800/01/01"[Date - Publication] : "2026/07/21"[Date - Publication])

**07_selection_failure_implementation**

("Cardiac Surgical Procedures"[Mesh] OR "cardiac surgery"[tiab] OR "cardiac surgical"[tiab] OR "heart surgery"[tiab] OR "open heart surgery"[tiab] OR "open-heart surgery"[tiab] OR "coronary artery bypass"[tiab] OR CABG[tiab] OR "valve surgery"[tiab] OR "off-pump coronary artery bypass"[tiab] OR OPCAB[tiab] OR "minimally invasive cardiac surgery"[tiab] OR "robotic cardiac surgery"[tiab] OR "aortic valve replacement"[tiab] OR "mitral valve surgery"[tiab] OR "thoracic aortic surgery"[tiab] OR "aortic root surgery"[tiab] OR "ascending aortic surgery"[tiab] OR "aortic arch surgery"[tiab] OR hemiarch[tiab] OR "type A aortic dissection"[tiab] OR "congenital heart surgery"[tiab] OR "congenital heart disease"[tiab] OR "ventricular septal defect"[tiab] OR "atrial septal defect"[tiab] OR "adult congenital heart disease"[tiab] OR "pediatric cardiac surgery"[tiab] OR "paediatric cardiac surgery"[tiab] OR "neonatal cardiac surgery"[tiab] OR "infant cardiac surgery"[tiab] OR "heart transplantation"[tiab] OR "cardiac transplantation"[tiab] OR "ventricular assist device"[tiab] OR LVAD[tiab]) AND ("fast-track failure"[tiab] OR "fast track failure"[tiab] OR "extubation failure"[tiab] OR "extubation readiness"[tiab] OR "extubation criteria"[tiab] OR "failure to extubate"[tiab] OR "delayed extubation"[tiab] OR ((predict*[tiab] OR "risk factor"[tiab] OR "risk factors"[tiab] OR implement*[tiab] OR feasib*[tiab] OR "protocol adherence"[tiab] OR "center experience"[tiab] OR "centre experience"[tiab]) AND ("early extubation"[tiab] OR "operating room extubation"[tiab] OR "on-table extubation"[tiab] OR "fast-track extubation"[tiab] OR "ultra-fast-track"[tiab] OR "ultra fast track"[tiab]))) AND english[Language] AND ("1800/01/01"[Date - Publication] : "2026/07/21"[Date - Publication])

**S2. Embase**

P = ('cardiac surg*':ti,ab,kw OR 'heart surg*':ti,ab,kw OR 'open heart surg*':ti,ab,kw OR 'coronary artery bypass':ti,ab,kw OR CABG:ti,ab,kw OR 'off-pump coronary artery bypass':ti,ab,kw OR OPCAB:ti,ab,kw OR 'valve surg*':ti,ab,kw OR 'aortic valve replacement':ti,ab,kw OR 'mitral valve surg*':ti,ab,kw OR 'minimally invasive cardiac surg*':ti,ab,kw OR 'robotic cardiac surg*':ti,ab,kw OR 'thoracic aortic surg*':ti,ab,kw OR 'aortic root surg*':ti,ab,kw OR 'ascending aortic surg*':ti,ab,kw OR 'aortic arch surg*':ti,ab,kw OR hemiarch:ti,ab,kw OR 'type A aortic dissection':ti,ab,kw OR 'congenital heart surg*':ti,ab,kw OR 'congenital heart disease':ti,ab,kw OR 'ventricular septal defect':ti,ab,kw OR 'atrial septal defect':ti,ab,kw OR 'adult congenital heart disease':ti,ab,kw OR 'pediatric cardiac surg*':ti,ab,kw OR 'paediatric cardiac surg*':ti,ab,kw OR 'neonatal cardiac surg*':ti,ab,kw OR 'infant cardiac surg*':ti,ab,kw OR 'heart transplant*':ti,ab,kw OR 'cardiac transplant*':ti,ab,kw OR 'ventricular assist device*':ti,ab,kw OR LVAD:ti,ab,kw)

O = (extubat*:ti,ab,kw OR reintubat*:ti,ab,kw OR 'mechanical ventilation':ti,ab,kw OR 'ventilation duration':ti,ab,kw OR 'mechanical ventilation time':ti,ab,kw OR 'time to extubation':ti,ab,kw OR 'respiratory complication*':ti,ab,kw OR 'pulmonary complication*':ti,ab,kw OR 'postoperative recovery':ti,ab,kw OR 'intensive care unit stay':ti,ab,kw OR 'ICU length of stay':ti,ab,kw)

A1 = ('ultra-fast-track':ti,ab,kw OR 'ultra fast track':ti,ab,kw OR UFTA:ti,ab,kw OR 'fast-track cardiac anesthe*':ti,ab,kw OR 'fast-track extubat*':ti,ab,kw OR 'operating room extubat*':ti,ab,kw OR 'operating-room extubat*':ti,ab,kw OR 'on-table extubat*':ti,ab,kw OR 'intraoperative extubat*':ti,ab,kw OR 'immediate extubat*':ti,ab,kw OR 'early extubat*':ti,ab,kw OR 'early tracheal extubat*':ti,ab,kw)

A2 = (((anesthe* OR anaesthe*) NEAR/3 (technique* OR management OR regimen* OR protocol*)):ti,ab,kw OR 'total intravenous anesthe*':ti,ab,kw OR TIVA:ti,ab,kw OR 'volatile anesthe*':ti,ab,kw OR 'balanced anesthe*':ti,ab,kw OR 'opioid-sparing':ti,ab,kw OR 'opioid sparing':ti,ab,kw OR 'multimodal analgesia':ti,ab,kw)

A3 = ('neuromuscular monitor*':ti,ab,kw OR 'quantitative neuromuscular monitor*':ti,ab,kw OR 'neuromuscular blockade':ti,ab,kw OR 'residual neuromuscular block*':ti,ab,kw OR 'train-of-four':ti,ab,kw OR 'train of four':ti,ab,kw OR ((neuromuscular OR 'muscle relaxant*') NEAR/3 revers*):ti,ab,kw OR sugammadex:ti,ab,kw OR neostigmine:ti,ab,kw)

A4 = ('enhanced recovery after surgery':ti,ab,kw OR 'enhanced recovery after cardiac surgery':ti,ab,kw OR ERAS:ti,ab,kw OR ERACS:ti,ab,kw OR 'enhanced recovery protocol*':ti,ab,kw OR 'enhanced recovery pathway*':ti,ab,kw OR 'fast-track protocol*':ti,ab,kw OR 'fast-track pathway*':ti,ab,kw OR 'extubation protocol*':ti,ab,kw)

A5 = ('regional analges*':ti,ab,kw OR 'regional anesthe*':ti,ab,kw OR 'fascial plane block*':ti,ab,kw OR 'erector spinae plane block*':ti,ab,kw OR 'paravertebral block*':ti,ab,kw OR 'parasternal block*':ti,ab,kw OR 'transversus thoracis plane block*':ti,ab,kw OR 'serratus anterior plane block*':ti,ab,kw OR 'intercostal nerve block*':ti,ab,kw)

A6 = ('high-flow nasal cannula':ti,ab,kw OR 'high flow nasal cannula':ti,ab,kw OR 'high-flow nasal oxygen':ti,ab,kw OR 'high flow nasal oxygen':ti,ab,kw OR HFNC:ti,ab,kw OR HFNO:ti,ab,kw OR 'noninvasive ventilation':ti,ab,kw OR 'non-invasive ventilation':ti,ab,kw OR NIV:ti,ab,kw OR CPAP:ti,ab,kw OR BiPAP:ti,ab,kw) AND (postextubat*:ti,ab,kw OR post-extubat*:ti,ab,kw OR extubat*:ti,ab,kw OR reintubat*:ti,ab,kw)

A7 = ('fast-track failure':ti,ab,kw OR 'fast track failure':ti,ab,kw OR 'extubation failure':ti,ab,kw OR 'extubation readiness':ti,ab,kw OR 'extubation criteria':ti,ab,kw OR 'failure to extubate':ti,ab,kw OR 'delayed extubation':ti,ab,kw OR ((predict* OR 'risk factor*' OR implement* OR feasib* OR 'protocol adherence' OR 'center experience' OR 'centre experience') AND ('early extubation' OR 'operating room extubation' OR 'on-table extubation' OR 'fast-track extubation' OR 'ultra-fast-track' OR 'ultra fast track')):ti,ab,kw)

F1 = P AND A1

F2 = P AND A2 AND O

F3 = P AND A3 AND O

F4 = P AND A4 AND O

F5 = P AND A5 AND O

F6 = P AND A6

F7 = P AND A7

**S3. Web of Science Core Collection**

#1 TS=("cardiac surg*" OR "heart surg*" OR "open heart surg*" OR "coronary artery bypass" OR CABG OR "off-pump coronary artery bypass" OR OPCAB OR "valve surg*" OR "aortic valve replacement" OR "mitral valve surg*" OR "minimally invasive cardiac surg*" OR "robotic cardiac surg*" OR "thoracic aortic surg*" OR "aortic root surg*" OR "ascending aortic surg*" OR "aortic arch surg*" OR hemiarch OR "type A aortic dissection" OR "congenital heart surg*" OR "congenital heart disease" OR "ventricular septal defect" OR "atrial septal defect" OR "adult congenital heart disease" OR "pediatric cardiac surg*" OR "paediatric cardiac surg*" OR "neonatal cardiac surg*" OR "infant cardiac surg*" OR "heart transplant*" OR "cardiac transplant*" OR "ventricular assist device*" OR LVAD)

#2 TS=(extubat* OR reintubat* OR "mechanical ventilation" OR "ventilation duration" OR "mechanical ventilation time" OR "time to extubation" OR "respiratory complication*" OR "pulmonary complication*" OR "postoperative recovery" OR "intensive care unit stay" OR "ICU length of stay")

#3 TS=("ultra-fast-track" OR "ultra fast track" OR UFTA OR "fast-track cardiac anesthe*" OR "fast-track extubat*" OR "operating room extubat*" OR "operating-room extubat*" OR "on-table extubat*" OR "intraoperative extubat*" OR "immediate extubat*" OR "early extubat*" OR "early tracheal extubat*")

#4 TS=(((anesthe* OR anaesthe*) NEAR/3 (technique* OR management OR regimen* OR protocol*)) OR "total intravenous anesthe*" OR TIVA OR "volatile anesthe*" OR "balanced anesthe*" OR "opioid-sparing" OR "opioid sparing" OR "multimodal analgesia")

#5 TS=("neuromuscular monitor*" OR "quantitative neuromuscular monitor*" OR "neuromuscular blockade" OR "residual neuromuscular block*" OR "train-of-four" OR "train of four" OR ((neuromuscular OR "muscle relaxant*") NEAR/3 revers*) OR sugammadex OR neostigmine)

#6 TS=("enhanced recovery after surgery" OR "enhanced recovery after cardiac surgery" OR ERAS OR ERACS OR "enhanced recovery protocol*" OR "enhanced recovery pathway*" OR "fast-track protocol*" OR "fast-track pathway*" OR "extubation protocol*")

#7 TS=("regional analges*" OR "regional anesthe*" OR "fascial plane block*" OR "erector spinae plane block*" OR "paravertebral block*" OR "parasternal block*" OR "transversus thoracis plane block*" OR "serratus anterior plane block*" OR "intercostal nerve block*")

#8 TS=(("high-flow nasal cannula" OR "high flow nasal cannula" OR "high-flow nasal oxygen" OR "high flow nasal oxygen" OR HFNC OR HFNO OR "noninvasive ventilation" OR "non-invasive ventilation" OR NIV OR CPAP OR BiPAP) AND (postextubat* OR post-extubat* OR extubat* OR reintubat*))

#9 TS=("fast-track failure" OR "fast track failure" OR "extubation failure" OR "extubation readiness" OR "extubation criteria" OR "failure to extubate" OR "delayed extubation" OR ((predict* OR "risk factor*" OR implement* OR feasib* OR "protocol adherence" OR "center experience" OR "centre experience") AND ("early extubation" OR "operating room extubation" OR "on-table extubation" OR "fast-track extubation" OR "ultra-fast-track" OR "ultra fast track")))

#10 = #1 AND #3

#11 = #1 AND #2 AND #4

#12 = #1 AND #2 AND #5

#13 = #1 AND #2 AND #6

#14 = #1 AND #2 AND #7

#15 = #1 AND #8

#16 = #1 AND #9

**S4. CINAHL**

P = "cardiac surg*" OR "heart surg*" OR "open heart surg*" OR "coronary artery bypass" OR CABG OR "off-pump coronary artery bypass" OR OPCAB OR "valve surg*" OR "aortic valve replacement" OR "mitral valve surg*" OR "minimally invasive cardiac surg*" OR "robotic cardiac surg*" OR "thoracic aortic surg*" OR "aortic root surg*" OR "ascending aortic surg*" OR "aortic arch surg*" OR hemiarch OR "type A aortic dissection" OR "congenital heart surg*" OR "congenital heart disease" OR "ventricular septal defect" OR "atrial septal defect" OR "adult congenital heart disease" OR "pediatric cardiac surg*" OR "paediatric cardiac surg*" OR "neonatal cardiac surg*" OR "infant cardiac surg*" OR "heart transplant*" OR "cardiac transplant*" OR "ventricular assist device*" OR LVAD

O = extubat* OR reintubat* OR "mechanical ventilation" OR "ventilation duration" OR "mechanical ventilation time" OR "time to extubation" OR "respiratory complication*" OR "pulmonary complication*" OR "postoperative recovery" OR "intensive care unit stay" OR "ICU length of stay"

A1 = "ultra-fast-track" OR "ultra fast track" OR UFTA OR "fast-track cardiac anesthe*" OR "fast-track extubat*" OR "operating room extubat*" OR "operating-room extubat*" OR "on-table extubat*" OR "intraoperative extubat*" OR "immediate extubat*" OR "early extubat*" OR "early tracheal extubat*"

A2 = ((anesthe* OR anaesthe*) N3 (technique* OR management OR regimen* OR protocol*)) OR "total intravenous anesthe*" OR TIVA OR "volatile anesthe*" OR "balanced anesthe*" OR "opioid-sparing" OR "opioid sparing" OR "multimodal analgesia"

A3 = "neuromuscular monitor*" OR "quantitative neuromuscular monitor*" OR "neuromuscular blockade" OR "residual neuromuscular block*" OR "train-of-four" OR "train of four" OR ((neuromuscular OR "muscle relaxant*") N3 revers*) OR sugammadex OR neostigmine

A4 = "enhanced recovery after surgery" OR "enhanced recovery after cardiac surgery" OR ERAS OR ERACS OR "enhanced recovery protocol*" OR "enhanced recovery pathway*" OR "fast-track protocol*" OR "fast-track pathway*" OR "extubation protocol*"

A5 = "regional analges*" OR "regional anesthe*" OR "fascial plane block*" OR "erector spinae plane block*" OR "paravertebral block*" OR "parasternal block*" OR "transversus thoracis plane block*" OR "serratus anterior plane block*" OR "intercostal nerve block*"

A6 = ("high-flow nasal cannula" OR "high flow nasal cannula" OR "high-flow nasal oxygen" OR "high flow nasal oxygen" OR HFNC OR HFNO OR "noninvasive ventilation" OR "non-invasive ventilation" OR NIV OR CPAP OR BiPAP) AND (postextubat* OR post-extubat* OR extubat* OR reintubat*)

A7 = "fast-track failure" OR "fast track failure" OR "extubation failure" OR "extubation readiness" OR "extubation criteria" OR "failure to extubate" OR "delayed extubation" OR ((predict* OR "risk factor*" OR implement* OR feasib* OR "protocol adherence" OR "center experience" OR "centre experience") AND ("early extubation" OR "operating room extubation" OR "on-table extubation" OR "fast-track extubation" OR "ultra-fast-track" OR "ultra fast track"))

F1 = P AND A1

F2 = P AND A2 AND O

F3 = P AND A3 AND O

F4 = P AND A4 AND O

F5 = P AND A5 AND O

F6 = P AND A6

F7 = P AND A7

**S5. Cochrane Library (CDSR and CENTRAL)**

**F1_direct_ufta_extubation (Cochrane Reviews: 1; CENTRAL Trials: 224; total: 225)**

((cardiac NEXT surg*) OR (heart NEXT surg*) OR (open NEXT heart NEXT surg*) OR "coronary artery bypass" OR CABG OR OPCAB OR (valve NEXT surg*) OR "aortic valve replacement" OR (mitral NEXT valve NEXT surg*) OR (minimally NEXT invasive NEXT cardiac NEXT surg*) OR (robotic NEXT cardiac NEXT surg*) OR (thoracic NEXT aortic NEXT surg*) OR (aortic NEXT root NEXT surg*) OR (ascending NEXT aortic NEXT surg*) OR (aortic NEXT arch NEXT surg*) OR "type A aortic dissection" OR (congenital NEXT heart NEXT surg*) OR (pediatric NEXT cardiac NEXT surg*) OR (paediatric NEXT cardiac NEXT surg*) OR (neonatal NEXT cardiac NEXT surg*) OR (heart NEXT transplant*) OR (cardiac NEXT transplant*) OR (ventricular NEXT assist NEXT device*) OR LVAD):ti,ab,kw AND ("ultra-fast-track" OR "ultra fast track" OR UFTA OR (fast-track NEXT cardiac NEXT anesthe*) OR (fast-track NEXT extubat*) OR (operating NEXT room NEXT extubat*) OR (on-table NEXT extubat*) OR (intraoperative NEXT extubat*) OR (immediate NEXT extubat*) OR (early NEXT extubat*) OR (early NEXT tracheal NEXT extubat*)):ti,ab,kw

**F2_anesthetic_techniques (Cochrane Reviews: 1; CENTRAL Trials: 880; total: 881)**

((cardiac NEXT surg*) OR (heart NEXT surg*) OR (open NEXT heart NEXT surg*) OR "coronary artery bypass" OR CABG OR OPCAB OR (valve NEXT surg*) OR "aortic valve replacement" OR (mitral NEXT valve NEXT surg*) OR (minimally NEXT invasive NEXT cardiac NEXT surg*) OR (robotic NEXT cardiac NEXT surg*) OR (thoracic NEXT aortic NEXT surg*) OR (aortic NEXT root NEXT surg*) OR (ascending NEXT aortic NEXT surg*) OR (aortic NEXT arch NEXT surg*) OR "type A aortic dissection" OR (congenital NEXT heart NEXT surg*) OR (pediatric NEXT cardiac NEXT surg*) OR (paediatric NEXT cardiac NEXT surg*) OR (neonatal NEXT cardiac NEXT surg*) OR (heart NEXT transplant*) OR (cardiac NEXT transplant*) OR (ventricular NEXT assist NEXT device*) OR LVAD):ti,ab,kw AND (((anesthe* OR anaesthe*) NEAR/3 (technique* OR management OR regimen* OR protocol*)) OR (total NEXT intravenous NEXT anesthe*) OR TIVA OR (volatile NEXT anesthe*) OR (balanced NEXT anesthe*) OR (short-acting NEXT anesthe*) OR (short NEXT acting NEXT anesthe*) OR "opioid-sparing" OR "opioid sparing" OR "opioid-free" OR "opioid free" OR "multimodal analgesia" OR remimazolam OR propofol OR sevoflurane OR desflurane OR remifentanil OR dexmedetomidine):ti,ab,kw AND (extubat* OR reintubat* OR "mechanical ventilation" OR "ventilation duration" OR "time to extubation" OR (respiratory NEXT complication*) OR (pulmonary NEXT complication*) OR "postoperative recovery" OR "intensive care unit stay" OR "ICU length of stay"):ti,ab,kw

**F3_neuromuscular_monitoring_reversal (Cochrane Reviews: 0; CENTRAL Trials: 60; total: 60)**

((cardiac NEXT surg*) OR (heart NEXT surg*) OR (open NEXT heart NEXT surg*) OR "coronary artery bypass" OR CABG OR OPCAB OR (valve NEXT surg*) OR "aortic valve replacement" OR (mitral NEXT valve NEXT surg*) OR (minimally NEXT invasive NEXT cardiac NEXT surg*) OR (robotic NEXT cardiac NEXT surg*) OR (thoracic NEXT aortic NEXT surg*) OR (aortic NEXT root NEXT surg*) OR (ascending NEXT aortic NEXT surg*) OR (aortic NEXT arch NEXT surg*) OR "type A aortic dissection" OR (congenital NEXT heart NEXT surg*) OR (pediatric NEXT cardiac NEXT surg*) OR (paediatric NEXT cardiac NEXT surg*) OR (neonatal NEXT cardiac NEXT surg*) OR (heart NEXT transplant*) OR (cardiac NEXT transplant*) OR (ventricular NEXT assist NEXT device*) OR LVAD):ti,ab,kw AND ((neuromuscular NEXT monitor*) OR (quantitative NEXT neuromuscular NEXT monitor*) OR "neuromuscular blockade" OR (residual NEXT neuromuscular NEXT block*) OR "train-of-four" OR "train of four" OR "neuromuscular reversal" OR "muscle relaxant reversal" OR sugammadex OR neostigmine):ti,ab,kw AND (extubat* OR reintubat* OR "mechanical ventilation" OR "ventilation duration" OR "time to extubation" OR (respiratory NEXT complication*) OR (pulmonary NEXT complication*) OR "postoperative recovery" OR "intensive care unit stay" OR "ICU length of stay"):ti,ab,kw

**F4_eras_pathways_protocols (Cochrane Reviews: 2; CENTRAL Trials: 90; total: 92)**

((cardiac NEXT surg*) OR (heart NEXT surg*) OR (open NEXT heart NEXT surg*) OR "coronary artery bypass" OR CABG OR OPCAB OR (valve NEXT surg*) OR "aortic valve replacement" OR (mitral NEXT valve NEXT surg*) OR (minimally NEXT invasive NEXT cardiac NEXT surg*) OR (robotic NEXT cardiac NEXT surg*) OR (thoracic NEXT aortic NEXT surg*) OR (aortic NEXT root NEXT surg*) OR (ascending NEXT aortic NEXT surg*) OR (aortic NEXT arch NEXT surg*) OR "type A aortic dissection" OR (congenital NEXT heart NEXT surg*) OR (pediatric NEXT cardiac NEXT surg*) OR (paediatric NEXT cardiac NEXT surg*) OR (neonatal NEXT cardiac NEXT surg*) OR (heart NEXT transplant*) OR (cardiac NEXT transplant*) OR (ventricular NEXT assist NEXT device*) OR LVAD):ti,ab,kw AND ("enhanced recovery after surgery" OR "enhanced recovery after cardiac surgery" OR ERAS OR ERACS OR (enhanced NEXT recovery NEXT protocol*) OR (enhanced NEXT recovery NEXT pathway*) OR (fast-track NEXT protocol*) OR (fast-track NEXT pathway*) OR (extubation NEXT protocol*)):ti,ab,kw AND (extubat* OR reintubat* OR "mechanical ventilation" OR "ventilation duration" OR "time to extubation" OR (respiratory NEXT complication*) OR (pulmonary NEXT complication*) OR "postoperative recovery" OR "intensive care unit stay" OR "ICU length of stay"):ti,ab,kw

**F5_regional_analgesia (Cochrane Reviews: 0; CENTRAL Trials: 289; total: 289)**

((cardiac NEXT surg*) OR (heart NEXT surg*) OR (open NEXT heart NEXT surg*) OR "coronary artery bypass" OR CABG OR OPCAB OR (valve NEXT surg*) OR "aortic valve replacement" OR (mitral NEXT valve NEXT surg*) OR (minimally NEXT invasive NEXT cardiac NEXT surg*) OR (robotic NEXT cardiac NEXT surg*) OR (thoracic NEXT aortic NEXT surg*) OR (aortic NEXT root NEXT surg*) OR (ascending NEXT aortic NEXT surg*) OR (aortic NEXT arch NEXT surg*) OR "type A aortic dissection" OR (congenital NEXT heart NEXT surg*) OR (pediatric NEXT cardiac NEXT surg*) OR (paediatric NEXT cardiac NEXT surg*) OR (neonatal NEXT cardiac NEXT surg*) OR (heart NEXT transplant*) OR (cardiac NEXT transplant*) OR (ventricular NEXT assist NEXT device*) OR LVAD):ti,ab,kw AND ((regional NEXT analges*) OR (regional NEXT anesthe*) OR (fascial NEXT plane NEXT block*) OR (erector NEXT spinae NEXT plane NEXT block*) OR (paravertebral NEXT block*) OR (parasternal NEXT block*) OR (transversus NEXT thoracis NEXT plane NEXT block*) OR (serratus NEXT anterior NEXT plane NEXT block*) OR (intercostal NEXT nerve NEXT block*)):ti,ab,kw AND (extubat* OR reintubat* OR "mechanical ventilation" OR "ventilation duration" OR "time to extubation" OR (respiratory NEXT complication*) OR (pulmonary NEXT complication*) OR "postoperative recovery" OR "intensive care unit stay" OR "ICU length of stay"):ti,ab,kw

**F6_postextubation_respiratory_support (Cochrane Reviews: 0; CENTRAL Trials: 146; total: 146)**

((cardiac NEXT surg*) OR (heart NEXT surg*) OR (open NEXT heart NEXT surg*) OR "coronary artery bypass" OR CABG OR OPCAB OR (valve NEXT surg*) OR "aortic valve replacement" OR (mitral NEXT valve NEXT surg*) OR (minimally NEXT invasive NEXT cardiac NEXT surg*) OR (robotic NEXT cardiac NEXT surg*) OR (thoracic NEXT aortic NEXT surg*) OR (aortic NEXT root NEXT surg*) OR (ascending NEXT aortic NEXT surg*) OR (aortic NEXT arch NEXT surg*) OR "type A aortic dissection" OR (congenital NEXT heart NEXT surg*) OR (pediatric NEXT cardiac NEXT surg*) OR (paediatric NEXT cardiac NEXT surg*) OR (neonatal NEXT cardiac NEXT surg*) OR (heart NEXT transplant*) OR (cardiac NEXT transplant*) OR (ventricular NEXT assist NEXT device*) OR LVAD):ti,ab,kw AND ("high-flow nasal cannula" OR "high flow nasal cannula" OR "high-flow nasal oxygen" OR "high flow nasal oxygen" OR HFNC OR HFNO OR "noninvasive ventilation" OR "non-invasive ventilation" OR NIV OR CPAP OR BiPAP):ti,ab,kw AND (postextubat* OR (post NEXT extubat*) OR extubat* OR reintubat*):ti,ab,kw

**F7_selection_failure_implementation (Cochrane Reviews: 0; CENTRAL Trials: 71; total: 71)**

((cardiac NEXT surg*) OR (heart NEXT surg*) OR (open NEXT heart NEXT surg*) OR "coronary artery bypass" OR CABG OR OPCAB OR (valve NEXT surg*) OR "aortic valve replacement" OR (mitral NEXT valve NEXT surg*) OR (minimally NEXT invasive NEXT cardiac NEXT surg*) OR (robotic NEXT cardiac NEXT surg*) OR (thoracic NEXT aortic NEXT surg*) OR (aortic NEXT root NEXT surg*) OR (ascending NEXT aortic NEXT surg*) OR (aortic NEXT arch NEXT surg*) OR "type A aortic dissection" OR (congenital NEXT heart NEXT surg*) OR (pediatric NEXT cardiac NEXT surg*) OR (paediatric NEXT cardiac NEXT surg*) OR (neonatal NEXT cardiac NEXT surg*) OR (heart NEXT transplant*) OR (cardiac NEXT transplant*) OR (ventricular NEXT assist NEXT device*) OR LVAD):ti,ab,kw AND ("fast-track failure" OR "fast track failure" OR "extubation failure" OR "extubation readiness" OR "extubation criteria" OR "failure to extubate" OR "delayed extubation" OR ((predict* OR risk OR implement* OR feasib* OR adherence) NEAR/5 (early NEXT extubat*)) OR ((predict* OR risk OR implement* OR feasib* OR adherence) NEAR/5 (fast-track NEXT extubat*)) OR ((predict* OR risk OR implement* OR feasib* OR adherence) NEAR/5 (operating NEXT room NEXT extubat*)) OR ((predict* OR risk OR implement* OR feasib* OR adherence) NEAR/5 (ultra-fast-track OR "ultra fast track"))):ti,ab,kw

**Record-management notes**

Records were unioned within each database before cross-database deduplication. Identifier-based deduplication used DOI, PMID, database accession number, and normalized title/first-author/year. Because many CENTRAL records lacked conventional bibliographic fields, exact normalized-title groups were subsequently adjudicated at report level. The final 90 records with unresolved preliminary classifications underwent two computer-assisted reassessments; 88 decisions agreed, and two discordant records were resolved in an additional adjudication pass. Full provenance, title-level adjudication, focused author audit, and aggregate author-confirmation records were retained in the revision archive.
