# Supplementary material for: Ultra-fast-track anesthesia in cardiac surgery: perioperative outcomes, patient selection, and implementation strategies—a narrative review
Source: Front Cardiovasc Med. 2026 Aug 12;13:1914025. doi: 10.3389/fcvm.2026.1914025 (PMC13507958; doi:10.3389/fcvm.2026.1914025)
Supplement: Supplementary file 2 [file Datasheet2.docx]

**Supplementary File 2: Comparative and Procedure-Specific Tables**

Supplementary Tables S5 and S6 provide the detailed study-level and procedure-specific evidence referenced in the main manuscript. They are supplied separately because each table spans multiple pages.

**Supplementary Table S5. Study-level quantitative summary of selected evidence on UFTA and operating-room extubation, organized by outcome domain.**

| **Study / year** | **Design, population, and procedure** | **UFTA definition and comparator** | **Ventilation / extubation** | **ICU LOS** | **Hospital LOS** | **Reintubation** | **Pulmonary complications** | **Mortality** | **Key limitations** |
| --- | --- | --- | --- | --- | --- | --- | --- | --- | --- |
| Santos et al., 2026 (adult meta-analysis)^40^ | Systematic review and meta-analysis; 20 studies involving nearly 800,000 adults | UFTE (operating-room extubation or extubation ≤1 h) vs FTE (extubation ≤6 h in ICU) | UFTE was protocol-defined as ORE or extubation ≤1 h; pooled ventilation duration NR | MD −12.27 h | MD −1.19 d | Overall: similar; MICS subgroup OR 0.26; propensity-matched cohorts: comparable | Pneumonia: similar; exact pooled estimate NR in the accessible report | 30-day mortality: OR 0.54; benefit not sustained in propensity-matched cohorts | Observational and database studies dominated; case mix, definition heterogeneity, and possible dataset overlap limit causal inference |
| Montero-Cruces et al., 2026 (CARDU-FAST)^41^ | Single-center randomized trial; 612 adults undergoing nonemergency cardiac surgery (306 vs 306) | UFT extubation in the operating theatre vs early ICU extubation | Prolonged intubation >24 h: 2.0% vs 7.5% (P=0.001); NIV: 5.6% vs 11.1% (P=0.013) | Shorter with UFT; value NR in the abstract | Shorter with UFT; value NR in the abstract | NR separately; included in the primary composite | Pneumonia NR separately; included in the primary composite | NR separately; all-cause mortality included in the primary composite | Single experienced center, inability to blind the pathway, and multiple secondary outcomes limit generalizability and superiority claims NIV 5.6% vs 11.1% was reported separately and was not classified as reintubation or a pulmonary complication. Primary composite endpoint: 6.5% vs 10.1% (P=0.105). |
| Etchill et al., 2025 (IMPROVE network)^15^ | Retrospective multicenter cohort; 79 hospitals and 95,982 direct-comparison patients (2,529 ORE and 93,453 early ICU extubations) | ORE vs extubation within 6 h of ICU arrival after on-pump surgery | Prolonged ventilation: 3.0% vs 2.0% | NR | NR | 5.2% vs 2.9% (P=0.003) | Pneumonia: 1.9% vs 1.1% (P<0.006) | In-hospital mortality: adjusted OR 1.34 (P<0.001) | ORE was uncommon, treatment selection and center experience were strong, and observational associations cannot establish harm |
| Teman et al., 2024 (STS database)^42^ | Retrospective national database study; 669,099 nonemergency adults across 1,069 hospitals; 36,298 received ORE | ORE vs FTE within 6 h for procedures with STS risk models | ORE: 36,298/669,099 (5.4%); other ventilation data NR | NR | Prolonged LOS: risk-adjusted results similar or better with ORE; estimates NR in the abstract | NR separately; reported only in a composite with reoperation for bleeding | NR | Operative mortality OR: CABG 0.54; AVR 0.43; MVR 0.48 | Only a selected minority received ORE; dynamic intraoperative eligibility was unavailable, and findings conflict with another large multicenter cohort |
| Carnero-Alcázar et al., 2023 (Spain)^8^ | Retrospective single-center cohort; 1,126 adults, including 331 propensity-matched pairs | UFT extubation before operating-room departure vs an FT goal of extubation within 6 h | ORE: 579 patients; matched intubation >24 h: 1.5% vs 5.4% (P=0.011) | 23.5 (22.0–46.0) vs 24.7 (21.5–62.9) h (P=0.015) | 6 (4–9) vs 7 (5–11) d (P=0.080) | 5/331 (1.5%) vs 10/331 (3.0%) (P=0.30) | Lung events: 2.4% vs 6.9% (P=0.011); pneumonia: 1.2% vs 3.9% (P=0.049) | In-hospital mortality: 1.8% vs 2.4% (P=0.77) | As-treated classification, clinical selection, and residual single-center confounding preclude causal interpretation |
| James et al., 2024 (isolated CABG)^38^ | Single-center retrospective STS cohort; 1,397 nonemergency isolated CABG patients; 414 propensity-matched pairs | ORE vs postoperative ICU extubation | ORE: 506/1,397 (36.2%) | 14 vs 20 h | 3 vs 5 d | No difference after matching; rates NR in the abstract | NR | 30-day mortality: no difference; rates NR in the abstract | Single experienced center, selected nonemergency CABG population, and residual confounding limit generalization |
| Xu et al., 2019 (pediatric randomized study)^10^ | Prospective randomized study; 194 children aged 6 months to 2 years with congenital heart disease (97 vs 97) | UFTA with attempted ORE vs conventional anesthesia and ICU extubation | Extubation time: 22.9 ± 3.5 vs 189.1 ± 31.2 min | 20.7 ± 6.5 vs 28.5 ± 4.2 h | 11.5 ± 3.0 vs 16.1 ± 2.4 d | 5/97 vs 6/97 | VAP: 3/97 vs 5/97; respiratory infection: 3/97 vs 4/97; CPAP use: 3/97 vs 3/97 | NR | Randomization and blinding methods were incompletely reported; selected age and lesion spectrum limit generalization, and the VAP significance claim was inconsistent with event counts |
| Fang et al., 2023 (pediatric VSD randomized trial)^11^ | Single-center randomized trial; 78 children randomized and 67 analyzed (33 vs 34) after thoracoscopic VSD closure | Attempted ORE vs postoperative ICU extubation | ORE: 33/33; mechanical ventilation: 0 vs 3.0 ± 3.7 h | 4.3 ± 2.5 vs 13.4 ± 4.4 h | 5.8 ± 0.8 vs 6.5 ± 1.2 d | 2/33 (6.1%) after ORE; comparator value NR | Respiratory complications: 0/33 vs 2/34 (5.7%) (P=0.492) | Perioperative mortality: 0 in both groups | Small selected cohort, postrandomization exclusions, and limited power for rare safety outcomes; reported cost direction was internally inconsistent |
| Parody Cuerda et al., 2023 (Spain) | Retrospective single-center era comparison; 1,498 adults undergoing mixed cardiovascular surgery | UFT era (ORE attempted in 785 patients) vs an earlier FT era (713 patients) | ORE: 699/785 (89%); other ventilation-time data NR | Shorter in unadjusted analysis; no independent association after adjustment; values NR | NR | NR | NR | Short-term survival higher in unadjusted analysis; no independent association after adjustment; rates NR | Nonrandomized era comparison with selection and institutional confounding. ^31^ |
| Tiganila et al., 2023 | Integrative review; six immediate-extubation outcome studies involving 2,109 adults, mainly after CABG or valve surgery | Immediate ORE vs early extubation where a comparator was available | Immediate ORE; reported extubation times ranged from 5–15 min to 0.23 ± 1.2 h; not pooled | Reported immediate-extubation means: 1.5 ± 0.6 to 4.3 ± 0.7 d; one RCT: 2.4 ± 0.78 vs 4 d (P<0.001) | NR | 0%-8% across studies; not pooled | Respiratory distress/pneumonia: 0.6%-2.5% where reported; not pooled | 0%-5.8% across studies; not pooled | No pooled meta-analysis; the review judged the evidence weak.^18^ |
| Stock et al., 2024 (Germany) | Single-center retrospective MICS cohort; 358 eligible patients and 297 successful on-table extubations | No conventional control; feasibility of six-hour monitored recovery and de-escalation was assessed | On-table extubation: 297/358 eligible patients | Median 22 h (IQR 20–24) to transfer from ICU/IMC to the ward | NR | 1/297 | One reintubation for hypercapnia; one later NIV episode for hypervolemia | No deaths among 297 patients during the reported hospitalization | Selected stable MICS patients and no conventional control group. ^43^ |
| Meier et al., 2025 (Europe) | Cross-sectional survey of 42 European pediatric cardiac surgical centers | Center-level ERAS and on-table-extubation practices; no patient-level comparator | Center definitions: on-table 13/42, ≤2 h 14/42, ≤6 h 15/42; no patient-level outcomes | NR | NR | NR | NR | NR | Survey of practice rather than a comparative clinical outcomes study. ^73^ |
| Bardia et al., 2022 (USA) | Double-blind placebo-controlled trial; 90 elective CABG and/or AVR patients | Sugammadex vs placebo after ICU arrival; not an ORE comparison | Drug-to-extubation time: 126 (84–274) vs 219 (121–323) min (P=0.01) | 2.0 (2.0–3.0) vs 2.0 (2.0–3.0) d (P=0.65) | 5.0 (4.0–6.0) vs 5.0 (4.0–6.0) d (P=0.77) | 0 vs 0 | No BiPAP or HFNC in either group; pulmonary complications otherwise NR | NR | Evaluates reversal in the ICU, not operating-room extubation or economic dominance. ^25^ |
| Salna et al., 2026 (aortic surgery)^44^ | Single-center descriptive cohort; 265 consecutive aortic operations, including root, Ross, hemiarch, total arch, reoperative, and circulatory-arrest procedures | ORE vs ICU extubation; 244/265 patients (92%) received ORE | ORE: 244/265 (92%); total ventilation: 5.8 (4.8–6.4) vs 15.1 (11.9–25.5) h | 7.5 (6.2–10.6) vs 35.6 (15.2–105.1) h | 3.1 (2.9–4.1) vs 5.8 (4.4–11.9) d | 0/244 vs 1/21 (5%) for respiratory failure | NR | 30-day mortality: 0/244 vs 0/21 | Single-center descriptive, nonrandomized study with strong institutional selection and a small ICU-extubation comparator; associations are not causal or broadly generalizable |
| Zayat et al., 2017 (adult LVAD)^36^ | Retrospective single-center pilot study; 53 adult LVAD implantations (50 HeartMate II and 3 HeartWare), with 13 successful UFTA patients and a propensity-matched comparison | In-theater extubation or extubation within 4 h after surgery; broader than strict within-1-h UFTA | Matched time to extubation: 1.2 ± 1.3 vs 42.3 ± 32.1 h; ORE in 5/13 UFTA patients | 60.2 ± 43.3 vs 153.1 ± 95.9 h (P=0.016) | 22.1 ± 9.5 vs 26.3 ± 14.9 d (P=0.055) | 1/13 (7.7%) vs 3/13 (23.1%) (P=0.125) | Pneumonia: 7.7% vs 46.5% (P=0.031); source count/percentage mismatch | 30-day mortality: 0 vs 1/13 (7.7%); longer-term survival not different | Small, selected, as-treated, single-center cohort with residual confounding; not a randomized test of strict UFTA The source reports pneumonia as 7/13 (46.5%) in the matched comparator, although 7/13 equals 53.8%; the published percentage was retained and the inconsistency is flagged. |
| Schnittman et al., 2017 (pediatric OHT)^51^ | Case series of four selected children after uncomplicated orthotopic heart transplantation | Immediate operating-room extubation; no comparator | ORE: 4/4; mild respiratory acidosis and hypercapnia resolved within 6-12 h | NR | NR | 0/4 | No pulmonary complication reported beyond transient acidosis/hypercapnia | 0/4 during reported follow-up; time window NR | Four cases, no control group, and highly selected uncomplicated transplantation; supports feasibility only |

Note: Values are reproduced as reported by the source studies and were not re-pooled. NIV was not classified as reintubation; composite outcomes were not disaggregated; mortality time windows were retained as reported; and outcomes outside the prespecified columns are not exhaustive. UFTA, ultra-fast-track anesthesia; ORE, operating-room extubation; ICU, intensive care unit; LOS, length of stay; NIV, noninvasive ventilation; NR, not reported in the accessible report; OR, odds ratio; MD, mean difference. Other procedure abbreviations are defined in the text.

**Supplementary Table S6. Mechanism-based procedural and physiologic considerations for UFTA candidacy.**

| **Procedure context** | **Features that may favor UFTA** | **Procedure-related modifiers requiring reassessment** | **Clinical interpretation** | **Reasons to defer immediate extubation** |
| --- | --- | --- | --- | --- |
| Off-pump OPCAB | No CPB; elective anatomy; stable hemodynamics, gas exchange, and hemostasis | The absence of CPB reduces inflammatory and rewarming burden, but conversion, bleeding, or instability removes this advantage. | One of the more favorable adult contexts, but real-time readiness and rescue capacity remain necessary. | Evidence is limited to selected OPCAB pathways; intraoperative high-dose vasopressors or other instability should defer extubation^17,31^ |
| On-pump CABG | Isolated elective procedure; limited CPB; prompt rewarming; stable rhythm and ventricular function after bypass | CPB duration, rewarming, rhythm, ventricular recovery, and vasoactive requirements determine whether the planned pathway remains appropriate. | Post-CPB physiology, not the CABG label alone, determines the final go/no-go decision. | Prolonged CPB time or postoperative low cardiac output should defer extubation; contemporary ORE experience is limited to selected CABG patients^32,38^ |
| Minimally invasive / robotic CABG | Limited incision; uncomplicated course; no conversion; stable monitored recovery pathway | Conversion to sternotomy, bleeding, prolonged operative or CPB time, and hemodynamic compromise negate the expected recovery advantage. | Rapid recovery may be feasible at experienced centers; conversion, bleeding, or instability overrides planned UFTA. | Perioperative instability, prolonged operative or CPB time, bleeding, conversion to sternotomy, or hemodynamic compromise^7^ |
| Aortic or mitral valve surgery | Uncomplicated isolated single-valve or minimally invasive procedure; limited CPB; secure hemostasis | Single-valve versus multivalve surgery, cross-clamp and CPB exposure, conduction disturbance, and ventricular recovery materially alter suitability. | Evidence is concentrated in selected single-valve or minimally invasive cohorts and should not be extrapolated to complex multivalve surgery. | Multiple valves, high bleeding risk^32^ |
| High-complexity combined cardiac surgery (e.g., multivalve or valve + CABG) | Only when procedural and CPB durations remain limited and recovery is uncomplicated | Cumulative operative trauma, prolonged CPB or cross-clamp exposure, hypothermia, coagulopathy, and myocardial dysfunction reduce predictability. | Strict UFTA should not remain a target when cumulative operative burden delays physiologic recovery. | Long CPB duration, postoperative instability^12^ |
| Root, ascending, or hemiarch aortic surgery | Experienced protocolized center; stable end-of-case neurologic, thermal, hemostatic, and hemodynamic recovery | Extent of repair, cerebral-protection strategy, circulatory arrest, temperature, coagulation, bleeding, and vasoactive requirements require end-of-case reassessment. | Recent single-center evidence supports feasibility in selected complex, reoperative, and circulatory-arrest cases, but it does not establish routine use across centers. | Unresolved neurologic concern, incomplete rewarming, coagulopathy, active bleeding, high vasoactive requirements, or inadequate rescue capacity^44,49^ |
| Total arch surgery / Acute aortic dissection | Selected case in an experienced protocolized program after complete rewarming, intact neurologic assessment, secure hemostasis, and stable hemodynamics | Circulatory arrest, cerebral-perfusion strategy, emergency status, coagulopathy, and neurologic uncertainty remain major modifiers of readiness. | Circulatory arrest is not an automatic exclusion in experienced-center reports, but evidence should not be generalized to emergency dissection or unresolved neurologic or hemostatic risk. | Emergency instability, unresolved neurologic concern, incomplete rewarming, coagulopathy, active bleeding, or high vasoactive requirements^44,48^ |
| Selected durable LVAD implantation | Adult INTERMACS 3–4 profile; stable device and right-ventricular function; secure hemostasis; low and non-escalating vasoactive support | Device flow, right-ventricular performance, pulmonary pressures, bleeding, perfusion, acid-base status, and support trajectory require end-of-case reassessment. | Small adult single-center cohorts support feasibility only; extubation endpoints may extend beyond the strict within-1-h UFTA definition. | INTERMACS 1 or shock, unresolved right-ventricular failure, open sternum, active bleeding or coagulopathy, escalating support, pulmonary edema, or inadequate perfusion^36,50^ |
| Pediatric orthotopic heart transplantation | Strictly selected uncomplicated transplant; stable graft and right-ventricular function; acceptable pulmonary vascular physiology; secure hemostasis | Graft performance, pulmonary vascular resistance, inotropic requirements, bleeding, gas exchange, acid-base status, and airway protection determine readiness. | Evidence is limited to a four-patient case series and supports feasibility, not routine use or comparative benefit. | Evidence is limited to four uncomplicated, highly selected transplant recipients; therefore, any primary graft dysfunction, pulmonary hypertension, escalating inotropes, hemodynamic instability, bleeding, gas-exchange abnormality, or airway concern should defer extubation^51^ |
| Older infants and children undergoing lower-complexity repair | Adequate weight; corrective lower-complexity procedure; no preoperative ventilation; uncomplicated CPB; stable pulmonary vascular physiology | Developmental age, STAT/RACHS context, CPB and cross-clamp exposure, residual lesions, pulmonary hypertension, airway risk, and vasoactive-inotropic requirements | Age and risk category guide reassessment but do not provide a validated UFTA threshold. | Complex neonatal anatomy^10,56,57^ |
| Neonatal and complex or single-ventricle pathways | Selected patient; no preoperative ventilation; adequate gestational age and weight; stable oxygenation and perfusion; short CPB; low and non-escalating vasoactive support | Stage-I shunt-dependent versus Glenn/Fontan physiology, pulmonary hypertension, residual lesions, open sternum, airway risk, and center rescue capacity | Norwood/HLHS should not routinely target UFTA; selected Glenn or Fontan patients may be considered after stage-specific reassessment. | Preoperative ventilation, low gestational age or weight, prolonged CPB, unstable shunt-dependent circulation, pulmonary hypertensive crisis, high vasoactive-inotropic requirements, hypoxemia, acidosis, residual lesions, or open sternum^54,56,61^ |
| Adult congenital heart disease (ACHD) | Selected anatomy; no major residual lesion; limited prior operative burden | Prior sternotomies, adhesions, residual anatomy, pulmonary vascular disease, and heterogeneous physiology limit procedure-label generalization. | Prior operations and heterogeneous anatomy require individualized reassessment. | Multiple prior surgeries^67^ |
| Low cardiac function (EF < 30%) | Exceptional cases with compensated physiology and stable separation from CPB | Dynamic ventricular recovery, lactate and acid-base status, pulmonary edema, rhythm, and support requirements are more informative than EF alone. | Low EF alone is insufficient, but unresolved low output or high support requirements should defer immediate extubation. | High-dose inotropic support required^31,50^ |
| Redo sternotomy or repeat cardiac surgery | Potentially feasible only after uncomplicated re-entry, limited adhesiolysis, secure hemostasis, and stable post-CPB physiology | Re-entry complexity, adhesiolysis, transfusion, operative duration, and the possibility of re-exploration increase rescue burden. | Repeat surgery is not an absolute contraindication, but the threshold for planned postoperative ventilation should be lower when re-entry or hemostasis is complex. | Dense adhesions, possible injury during re-entry, transfusion, prolonged operative time, or re-exploration risk^52^ |
| Postoperative mechanical circulatory or high-dose vasoactive support | No ongoing support requirement; stable native or device-supported hemodynamics in a highly selected pathway | The type and trajectory of support, ventricular recovery, perfusion, pulmonary edema, and acid-base status determine readiness. | The presence of support is a marker for intensified reassessment, not an automatic permanent exclusion; published feasibility is confined to highly selected experienced-center cohorts. | IABP or ECMO, unresolved ventricular failure, escalating or high-dose vasoactive support, pulmonary edema, acidosis, or inadequate perfusion^31,36^ |

Note: This table distinguishes operating-room extubation, UFTA (operating-room extubation or extubation within 1 h), fast-track extubation (1-6 h), and delayed extubation (>6 h). Extubation beyond 1 h after surgery is more appropriately classified as fast-track or early extubation rather than strict UFTA. The categories are illustrative and should guide dynamic reassessment rather than serve as absolute contraindications. Failure to meet extubation-readiness criteria should prompt planned postoperative ventilation without being regarded as pathway failure. The reasons-to-defer column is a narrative clinical synthesis of the cited evidence and perioperative principles, not a set of validated procedure-specific criteria.
